# Estimating the nationwide transmission risk of measles in US schools and impacts of vaccination and supplemental infection control strategies

**DOI:** 10.1186/s12879-020-05200-6

**Published:** 2020-07-11

**Authors:** Parham Azimi, Zahra Keshavarz, Jose Guillermo Cedeno Cedeno Laurent, Joseph G. Allen

**Affiliations:** Department of Environmental Health, Harvard T. H. Chan School of Public Health, Boston, USA

**Keywords:** Measles, Nationwide transmission risk, US schools, Vaccination, Control strategies

## Abstract

**Background:**

The spread of airborne infectious diseases such as measles is a critical public health concern. The U.S. was certified measles-free in 2000, but the number of measles cases has increased in recent years breaking the record of the nationwide annual number of cases since 1992. Although the characteristics of schools have made them one of the most vulnerable environments during infection outbreaks, the transmission risk of measles among students is not completely understood. We aimed to evaluate how three factors influence measles transmission in schools: personal (vaccination), social (compartmentalizing), and building systems (ventilation, purification, and filtration).

**Methods:**

We used a combination of a newly developed multi-zone transient Wells-Riley approach, a nationwide representative School Building Archetype (SBA) model, and a Monte-Carlo simulation to estimate measles risk among U.S. students. We compared our risk results with the range of reported transmission rates of measles in school outbreaks to validate the risk model. We also investigated the effectiveness of vaccination and ten supplemental infection control scenarios for reducing the risk of measles transmission among students.

**Results:**

Our best nationwide estimate of measles transmission risk in U.S. schools were 3.5 and 32% among all (both unvaccinated and immunized) and unvaccinated students, respectively. The results showed the transmission risk of measles among unvaccinated students is > 70 times higher than properly immunized ones. We also demonstrated that the transmission risk of measles in primary schools (assuming teacher self-contained classrooms) is less than secondary schools (assuming departmentalized systems). For building-level interventions, schools with ductless-with-air-filter and ductless-without-air-filter systems have the lowest and highest transmission risks of measles, respectively. Finally, our simulation showed that infection control strategies could cut the average number of infected cases among all students in half when a combination of advanced air filtration, ventilation, and purification was adopted in the modeled schools.

**Conclusions:**

Our results highlight the primary importance of vaccination for reducing the risk of measles transmission among students. Yet, additional and significant risk reduction can be achieved through compartmentalizing students and enhancing building ventilation and filtration systems.

## Background

The spread of airborne infectious diseases is a global public health concern. Measles is an airborne viral respiratory illnesses that remains a significant cause of death worldwide and imposes an extreme economic burden on communities and families despite the availability of a safe and cost-effective vaccine [1]. It is estimated that approximately 110,000 people, mostly children under the age of 5 years, died from measles globally in 2017 [2]. High incidence of measles among healthy children gives rise to considerable morbidity [3], which, in turn, makes the role of school environments crucial in the spread of measles in the community. It is shown that the transmission of respiratory illnesses such as measles in schools leads to large excesses in expenses associated with healthcare, absence from school, and reduction in students’ productivity [4–7].

Concerns about measles is a public health issue which dates back many centuries. A brief history of measles tells us a Persian physician published one of the first written accounts of measles disease in the ninth century; in 1757 a Scottish doctor, Francis Home, demonstrated that measles is caused by an infectious agent; in 1912 measles became a nationally notifiable disease in the U.S.; and finally in 1963, the measles vaccine became available in the United States [8]. Since then, the annual number of measles cases in the U.S. decreased drastically from ~ 450,000 cases just before the introduction of the measles vaccine to less than 100 cases in 2000, when the U.S. was certified measles-free [9, 10]. The measles vaccine coverage in the U.S. has remained relatively constant since the Vaccines for Children program began in 1994 [11]. However, the number of annual measles cases has increased in recent years. In 2019, 1282 individual cases of measles have been confirmed in 31 states, which was the greatest number of cases reported in the U.S. since 1992 [12]. The majority of the cases have been among unvaccinated individuals. This demonstrates, primarily, the danger that unvaccinated cohorts deliver to the community including to infants younger than 12 months old, pregnant women, and individuals allergic to the vaccine or who haveweakened immune systems and thereforewho should not get vaccinated. Furthermore, this demonstrates the importance of understanding the transmission mechanisms of the measles virus in the built environment and adopting efficient control strategies to reduce the infection risk of measles among susceptible individuals. It is particularly important to investigate measles transmission among students at schools given that educational institutes are considered one of the most vulnerable environments in transmission of airborne infectious disease due to the extensive amount of time that students regularly spend in schools and the high levels of interactions among schoolchildren that occur in these environments [13–15].

The airborne transmission of measles in indoor environments such as schools and associated risk of infection presented to susceptible occupants are governed by several complex physical, biological, and epidemiological processes. Mathematical models have long been used to predict the transmission risk of airborne infectious diseases in the built environment. Epidemic modeling approaches such as susceptible-infector-susceptible (SIS) [16], susceptible-infectious-recovered (SIR) [17] competing-risks [18, 19], neural network [20], and Reed-Frost [21, 22] models are used to describe the progression of a disease in a population, although it is shown that these models alone cannot explain the spread of airborne infectious diseases such as measles in indoors environments [22]. Therefore, epidemic models are usually combined with other mathematical approaches to predict the risks associated with indoor spaces such as airplanes, hospitals [3], schools [17], residences [23], and healthcare facilities [24].

Another mathematical approach is the dose-response model, which has been adopted to estimate the airborne infection risks associated with the dose of infectious agents delivered to upper and lower respiratory tracts of a susceptible individual. This model requires the use of an underlying fate and transport model such as Markov chains, computational fluid dynamics (CFD), multi-zone mass balance models, or combinations of these methods to estimate the number of delivered viable pathogens to the infection sites in the respiratory tracts [25–30]. The complexity of combination of the dose-response approach and mathematical transport models limits the application of this simulation approach to only well-described indoor environments and diseases such as influenza.

Among all mathematical risk models, the Wells-Riley model is the most common approach [31], which was introduced originally by Wells [32] and Riley et al. [33]. The Wells–Riley model has been extensively used in analyzing ventilation strategy and its association to airborne infections in indoor environments and considered as a valid approach for estimating the transmission risk of airborne infectious diseases [34]. The Wells-Riley model is a relatively simple model that can be deployed with less required information regarding the characteristics of a desired space or disease in comparison to other mathematical risk approaches such as the dose-response model. Despite the validity and popularity of the Wells-Riley model, only a few studies used this approach to estimate the transmission risk of measles in an educational setting and all of them modeled the setting as one well-mixed indoor space [33, 35, 36]. This makes the results of the risk models less accurate, particularly for large and complex indoor environments. A limited number of studies used developed multi-zone versions of the Wells-Riley model for other indoor spaces such as hospitals and multifamily residential buildings [31, 37, 38], which demonstrates the potential capability of a multi-zone version of the model for predicting the infection risk of airborne infectious diseases such as measles in other complex indoor environments such as schools.

There is also a gap in understanding the efficiency of various infection control strategies beyond vaccination in reducing the transmission risk of airborne pathogens in indoor environments and particularly for lowering measles risk in schools. Many studies have demonstrated the most effective control strategy against measles is adequate vaccination [39–41] meaning children get two doses of vaccine, starting with the first dose at 12 through 15 months of age, and the second dose at 4 through 6 years of age and teens and adults should also be up to date on their vaccinations [42]. However, despite high vaccination records, explosive measles outbreaks may occur in school environments due to (i) the fact that a portion of adequately immunized individuals remains susceptible to measles viruses, (ii) high interaction and contact rates among students, or (iii) inadequate immunity from vaccinations at younger ages [22]. Heating, ventilation, and air-conditioning (HVAC) systems and air purifiers are shown to have a significant positive impact on reducing the transmission of measles in various indoor environments by enhancing filtration, ventilation and purification rates [43–47]; but, their bio-aerosol removal effectiveness for a typical school setup is understudied.

Moreover, in the best of our knowledge, all of the existing mathematical risk models have been applied to an indoor environment with specific building, HVAC, and occupational characteristics. These model outcomes normally could not be the representative of infection risk in a certain type of environment in a region or nationwide. Consequently, standard developers and policymakers are reluctant to use the existing model results for establishing new guidelines to control the transmission risk of infectious airborne diseases including measles in vulnerable indoor environments such as schools. Developing a nationwide representative School Building Archetype (SBA) model and combining it with a proper mathematical risk approach would form a powerful transmission risk tool that helps to fulfill this shortcoming in the knowledge of airborne infectious disease transmissions.

This research work primarily aims to investigate the transmission risk of measles in U.S. schools. Herein, we combined a newly developed multi-zone transient Wells-Riley model with a nationwide representative SBA model to estimate the transmission risk of measles in primary and secondary educational institutions in the U.S. and evaluate the performance of several control strategies for reducing measles infection risk.

## Methods

### Developing Wells-Riley model for several microenvironments

The Wells-Riley model was developed originally by Wells [32] and Riley et al. [33] to estimate the probability of airborne transmission of an infectious agent indoors. Rudnick and Milton [35] developed a new derivation of the Wells-Riley model in which the probability of infection transmission (*P*_*infection*_) in a well-mixed indoor space can be estimated using Eq. 1.

1 $$ {P}_{infection}=\frac{Number\ of\ Infected\ Cases}{Number\ of\ Susceptible\ Individuals}=1-{e}^{-\mu } $$

In this risk model, μ is the number of quantum of infection or ‘quanta’ breathed by a susceptible person. It is important to notice that quanta is not an actual physical unit; rather, it is a hypothetical infectious dose unit, which is typically back-calculated from observational epidemiological studies. Wells suggested the average risk of becoming infected after exposure to one quanta is 63% (i.e. 1 − *e*^−1^), which is essentially a 63% infectious dose, ID_63_ [32].

Herein, we considered three microenvironments or spaces within a typical U.S. school building to estimate the transmission risk of infectious aerosols between students (i.e. droplet nuclei containing measles viruses in this research work) after *one* index case (infector) enters the school, including:

(i) Infector’s classroom: the school classroom, where the index case (infector) spends most of their time
(ii) Recirculation spaces: the spaces within a typical school building (e.g. classrooms, labs, and hallways), where generated infectious bio-aerosols would reach there *only* via HVAC system air recirculation from the infector’s classroom
(iii) Common spaces: spaces other than the infector’s classroom, where the index case physically presents for a considerable amount of time and interacts with other students

In this case, the average number of quanta breathed by susceptible students during a typical school day ($\overline{\mu}\Big)$ can be estimated using Eq. 2.

2 $$ \overline{\mu}=\frac{1}{N_{total}}\times \overline{p}\times \sum \limits_i{\int}_0^{{\overline{t}}_i}{N}_i\left(\tau \right).{C}_{quanta,i}\left(\tau \right) d\tau $$

*N*_*total*_: Total number of students in the schools during the infection period.

$\overline{p}$: Average breathing rate of one student (m^3^ / hour).

${\overline{t}}_i$: Average time that students spend in space *i* (hour).

*N*_*i*_(*τ*): Number of students in space *i* as a function of time.

*C*_*quanta*,*i*_(*τ*): Concentration of quanta in space *i*, *τ* hours after the index case enters the space (quanta / m^3^).

We made several simplifications for estimating the average number of breathed quanta by susceptible students including:

(i) Students stay continuously in each space during an exposure period
(ii) The number of students in each space remains constant during an exposure period
(iii) Other transmission pathways of measles viruses such as direct contact or fomite are ignored

It is noticeable that the potential impacts of these assumptions on the risk transmission results were taken into the account indirectly by back-calculating the quanta generation rate from actual measles outbreak cases in two U.S. schools (i.e. one elementary and one high school) with different interaction patterns among students. The back-calculation process is described in detail in the “back calculating quanta generation rate” section.

The concentration of quanta in the infector’s classroom and the common space *τ* hours after presence of the index case, *C*_*qunata*,*j*_, can be estimated using Eq. 3, by solving a well-mixed mass balance equation for each of these two spaces as described in Appendix A.

3 $$ {C}_{quanta,j}\left(\tau \right)=\frac{Iq}{V_j{K}_{total,j}}\left(1-{e}^{-{K}_{total,j}\tau}\right) $$

*I*: Number of index cases.

*q*: Quanta generation rate (quanta / hour).

*V*_*j*_: Volume of space *j* – either infector’s classroom or common space – (m^3^).

*K*_*total*,*j*_: Total removal rate of measles viruses in space *j* – either infector’s classroom or common space – (per hour).

In this model, we assumed the recirculated air is the *only* pathway that the infectious particles can reach the recirculation space from the infector’s classroom. We adopted a discrete time-varying mass balance to estimate the concentration of quanta in the recirculation space. The concentration of quanta in the recirculation space at each time step [*C*_*quanta*,*recir*_(*τ*_*n*_)] was estimated using Eq. 4. The steps for developing Eq. 4 are shown in Appendix A.

4 $$ {C}_{quanta, recir}\left({\tau}_n\right)=\Delta  \tau \left[-{K}_{total, recir}{C}_{quanta, recir}\left({\tau}_{n-1}\right)\left(\frac{Q_{return, class}{f}_{recir}{f}_{runtime}\left(1-{\eta}_{filter}\right)}{V_{recir}}\times \frac{Q_{supply, recir}}{Q_{supply, total}}\right){C}_{quanta, class}\left({\tau}_{n-1}\right)\right]+{C}_{quanta, recir}\left({\tau}_{n-1}\right) $$

*∆τ*: Time step interval, which is considered one minute in this model (hour).

*K*_*total*,*recir*_: Total removal rate of measles viruses in recirculation space (per hour).

*Q*_*return*,*class*_: Return airflow rate of the infector’s classroom (m^3^/hour).

*f*_*recir*_: Fraction of recirculated air volume to total airflow capacity of HVAC system.

*f*_*runtime*_: Runtime fraction of HVAC system.

*η*_*filter*_: Removal efficiency of HVAC air filter.

*Q*_*supply*,*recir*_: Supply airflow rate of the recirculation space (m^3^/hour).

*Q*_*supply*,*total*_: Total supply capacity of HVAC system (m^3^/hour).

*C*_*quanta*,*class*_(*τ*_*n*_): Concentration of quanta in infector’s classroom (quanta / m^3^).

*V*_*recir*_: Volume of recirculation space (m^3^).

The total removal rate of measles viruses in space *i* (*K*_*total*,*i*_) – infector’s classroom, recirculation space, and common space – was estimated by summing the rates of five infection removal mechanisms as shown in Eq. 5.

5 $$ {K}_{total,i}={\lambda}_{infiltration,i}+{K}_{deposition,i}+{K}_{ventilation,i}+{K}_{filtration,i}+{K}_{purification,i} $$

*λ*_*infiltration*,*i*_: Natural air ventilation rate or infiltration air exchange rate in space i (per hour).

*K*_*deposition*,*i*_: Deposition rate of measles particles in space i (per hour).

*K*_*ventilation*,*i*_: Mechanical ventilation rate of HVAC system in space i (per hour).

*K*_*filtration*,*i*_: Infectious particle removal rate due to filtration in space i (per hour).

*K*_*purification*,*i*_: Removal rate of infectious particles by standalone air handling units (AHU) or air purifiers in space i (per hour).

The infiltration air exchange rate (*λ*_*infilteration*_) of a typical school in the U.S. was estimated to be 0.31 per hour ranging between 0.12 and 0.49 per hour based on the U.S. Department of Energy (DOE) commercial reference-building models for educational buildings [48]. The DOE’s commercial reference-building model represents approximately two-thirds of the national building stock in the U.S.

Deposition (*K*_*deposition*_) and filtration (*K*_*filtration*_) rates of infectious particles in indoor environments depend on the distribution of measles viruses in different bio-aerosol size ranges. We are not aware of any study that reports the size distribution of measles viruses in indoor aerosols. Several studies have reported the size distribution of dry powder measles vaccine for aerosol delivery [49–51]; however, there is no evidence that the distribution of measles in the powder vaccine is similar to measles virus size distribution in humans-generated bio-aerosols. In this study, because of the lack of a reliable source, we assumed that the size distribution of measles viruses in indoor bio-aerosols is similar to influenza viruses. This assumption is based on the fact that both diseases are airborne viral respiratory infections with similar virus sizes ranging between 80 and 120 nm [52] and 100–200 nm [53] for influenza and measles viruse, respectively.

As we assumed a similar size distribution for measles and influenza viruses, their estimated deposition rate and air filter removal efficiencies will be similar and equal to the reported values in Azimi and Stephens [54]. Azimi and Stephens estimated the deposition rate of influenza viruses to range between 1 and 2.3 per hour and the average removal efficiency (*η*_*filter*_) of air filters for particles containing influenza viruses to be between 10.5 and 99.9% for filters with minimum efficiency reporting value (MERV) of 4 and high-efficiency particulate air (HEPA) filters, respectively, as demonstrated in Table 1.

**Table 1:** Infectious-particle-size-weighted filtration efficiency for a range of HVAC air filters [54]

Filter Type	Range	Mean
MERV 4	7.7–12.7%	10.5%
MERV 7	35.5–47.4%	42.2%
MERV 13	81.6–89.2%	85.9%
MERV16	95.0%	95.0%
HEPA	99.9%	99.9%

The particle removal rate due HVAC filtration (*K*_*filtration*,*i*_) for each space was estimated from Eq. 6.

6 $$ {K}_{filtration,i}=\frac{f_{runtime}\times {f}_{recir}\times {\eta}_{filter}\times {Q}_{return,i}}{V_i} $$

*Q*_*return*,*i*_: Return air flow rate of HVAC system in space i (m^3^/hour).

*V*_*i*_: Volume of space i (m^3^).

Similarly, the mechanical ventilation rate of HVAC systems (*K*_*ventilation*,*i*_) in space *i* can be estimated from Eq. 7.

7 $$ {K}_{ventilation,i}=\frac{f_{runtime}\times \left(1-{f}_{recir}\right)\times {Q}_{return,i}}{V_i} $$

Finally, the removal efficiency of measles viruses by an air purifier in space i (*K*_*purification*,*i*_) was estimated from Eq. 8.

8 $$ {K}_{purification,i}=\frac{f_{runtime, AP,i}\times CAD{R}_{AP,i}}{V_i} $$

*f*_*runtime*,*AP*_: Runtime fraction of air purifier in space i.

*CADR*_*AP*,*i*_: Clean air delivery rate of air purifier in space i (m^3^/hour).

The CADR of an air purifier is usually estimated by multiplying the air delivery rate and air filter removal efficiency of the air purifier. As most of air purifiers using HEPA filters with removal efficiencies of more than 99% for all particle sizes and types, we assumed the clean air delivery rate of an air purifier, when challenged with bio-aerosols containing measles viruses, is similar to the factory reported CADR of the air purifier.

### Estimating the number of susceptible individuals based on vaccination coverage and age

The transmission risk of measles among occupants of an indoor environment depends on the number of susceptible individuals in that cohort, which is estimated using a variety of approaches before, during, and after a measles outbreaks in existing studies. The simplest approach is to assume that everyone is susceptible [3], which is not a realistic approach, particularly for a population with a high vaccination coverage. The most reliable approach is to measure the level of Immunoglobulin G (IgG) antibodies against measles in the occupants’ blood [55, 56]; however, this approach is costly and time-consuming making it hardly available for every measles outbreak. As another approach, Riley et al. suggested to assume that the total number of susceptible individuals is equal to the number of infected cases at the end of an outbreak assuming the outbreak would stop only after all susceptible individuals were infected [33]. This approach by Riley et al. ignores the fact that the number of infected cases and accordingly their estimate of susceptible individuals would change if a different infection control strategy was deployed during the outbreak.

A proper approach for estimating susceptibility in a cohort is based on the vaccination coverage and age of the individuals in that cohort. Several studies have suggested that measles vaccine efficacy is not flawless, and a small portion (i.e., less than 10%) of vaccinated individuals would always remain susceptible to the disease [57–60]. Landen et al. assumed a 1 and 5% vaccination failure among students receiving two doses and one dose of vaccine, respectively, and 100% susceptibility for non-vaccinated students during the 1996 measles outbreak in Alaska, U.S. [61]. Choi et al. assumed infants less than 6 months old are immune to measles through maternal antibody, cohorts between 6 months and 14 years old are 100, 10 and 1% susceptible if they had received no vaccine, 1 dose of vaccine, and 2 doses of vaccine, respectively, and 5 and 2% of immunized young students between 14 and 24 years old and adults older than 25 years old are susceptible to measles, respectively [62].

In this study, we estimated the percentage of susceptible individuals in the U.S. schools based on age and vaccination coverage similar to Choi et al. [62]. We assumed elementary students less than 14 years old are 100, 10% or 1%, susceptible to measles viruses if they were not vaccinated or had one dose or two doses of the vaccine, respectively, before the outbreak. We also assumed 5% of secondary students between 14 and 18 years old are susceptible to the measles viruses, if they received one or two doses of measles vaccine before an outbreak. We also compare our assumption for number of susceptible people with the reported number of infected cases during actual measles outbreaks in primary and secondary schools in developed countries to verify our assumptions.

### Back calculating quanta generation rate

Quanta generation rate (q) in the Wells-Riley model is a critical parameter back-calculated from observational epidemiological studies indicating the contagiousness of an airborne pathogen. It is important to note that for any new derivation of the Wells-Riley model a new associated quanta generation rate should be back-calculated for a desired pathogen, reflecting the assumptions used in developing the new risk model. For example, Riley et al. estimated the quanta generation rate of measles between 480 and 5589 quanta per hour from an outbreak in an elementary school in New York using a steady state Well-Riley model [33]. Later, Rudnick and Milton reported a quanta generation rate of 570 quanta per hour using a set of new assumptions for the risk model [35] and Chen et al. back-calculated a quanta generation rate of 128 quanta per hour using a transient derivation of the risk model [3] for the exact same measles outbreak in the New York elementary school studied firstly by Riley et al.

In this research work, we relied on two well-known studies that have described measles outbreaks in primary and secondary schools in the U.S. to back-calculate the quanta generation rate of measles for our newly developed risk model [22, 33]. These studies were selected because they reported the detailed characteristics of measles outbreaks in the schools. Both studies include information on the school floor plan, HVAC system operation time and characteristics, index case activity patterns, and vaccination records of students before and during the outbreaks. We selected separate case studies for primary and secondary schools because model parameters, such as students’ interaction, susceptibility, and inhalation rate, are varied among students under 14 years old and between 14 and 18 years old attending primary and secondary schools, respectively.

Similar to the developed risk model, we divided the school environment into three spaces or microenvironments including the infector’s classroom, recirculation spaces, and common spaces. Table 2 demonstrates our primary estimates and ranges for the risk model parameters. Most of the model variables were reported directly in Riley et al. and Chen et al. studies; however, some of the parameters were not reported during the outbreaks. In these cases, we considered a range for the model variables and chose a ‘best’ or ‘primary estimate’ for each model parameter reflecting our finest estimations of that variable as shown in Table 2.

**Table 2:** Summary of outbreak characteristics in primary and secondary representative schools used in quanta generation rate (q) back-calculation process

Parameter	Primary School Best-Estimate [Range]	Secondary School Best-Estimate [Range]	Reference
No. of enrolled students during outbreaks	868	1873	Literature^a^
No. of first generation infected cases	28	69	Literature^a^
No. of index case/s	1	1	Literature^a^
Infection period in school (day)	3	4	Literature^a^
Portion of unvaccinated students	3.3%	0.3%	Literature^a^
Portion of students with 1-dose vaccination	96.7%	70.9%	Literature^a^
Portion of students with 2-dose vaccination	0.0%	28.8%	Literature^a^
No. of students in infector’s classroom	24	30	Literature^a^
No. of students in recirculation area	592	1843	Literature^a^
No. of students in common area	664	1873	Literature^a^
Average time spent in classroom/s (min)	280 [270–290]^b^	340	Literature^a^
Average time spent in recirculation area (min)	280 [270–290]^b^	340	Literature^a^
Average time spent in common area (min)	20 [10–30]^b^	70	Literature^a^
HVAC system runtime fraction	1	0.768	Literature^a^
Recirculated air fraction	0.438	0.05	Literature^a^
Supply airflow rate of one classroom (m^3^/min)	28.3	8.5	Literature^a^
Total HVAC system capacity (m^3^/min)	1019.4	595	Literature^a^
Air filter removal efficiency (%)	12	12^c^ [10.5–42.2]	Literature^d^
Occupancy density of classroom (m^2^/person)	4 [3–5]	4 [3–5]	DOE^e^
Volume of recirculation area (m^3^)	13,832 [10374–17,290]	33,600 [25200–42,000]	Estimated^f^
Occupancy density of common area (m^2^/person)	1.39 [1.04–1.74]	1.39 [1.04–1.74]	DOE^e^
Inhalation rate (m^3^/day)	12.96[11.34–14.53]	15.53 [13.93–17.45]	EPA^g^
Deposition rate of measles bio-aerosols (1/h)	1.7 [1.0–2.7]	1.7 [1.0–2.7]	Literature^d^
Natural ventilation rate (1/h)	0.31 [0.12–0.49]	0.31 [0.12–0.49]	DOE^h^

^a^Based on the information reported in Riley et al. (1978) and Chen et al. (1989) case studies [22, 33]

^b^Assuming similar lunchtime as Chen et al. (1989) [22] case study ± 50%

^c^For the primary estimate we considered the reported removal efficiency in Riley et al. (1978) [33]

^d^Azimi and Stephens (2013), Table 4; assuming MERV4 and MERV 7 for the air filters [54]

^e^U.S. Department of Energy commercial reference building models of the national building stock report, [48]; the average density of students in educational buildings (±25%)

^f^For the elementary school estimated based on the HVAC total capacity versus supply air flow of each classroom and for the high school calculated based on the of occupancy density of classrooms and the school’s floor plan

^g^U.S. Environmental Protection Agency (EPA), [63]; Interpolated from the reported inhalation rates of children in various age ranges in the Exposure Factors Handbook: 2011 Edition, Table 6.23

^h^U.S. Department of Energy commercial reference building models of the national building stock, [48]; Table A-2, primary and secondary education buildings

In Appendix B, the process for culling the model parameters from the case study articles and other listed references is explained in detail and the results are summarized in Table S1 and Figure S1. It is noticeable that because we back-calculated the quanta generation rate from actual epidemiology studies, any simplification that we deployed during the model development and variable estimation will be considered automatically in the quanta generation estimates. We also determined the boundaries of the quanta generation rates for the primary and secondary schools based on the ranges of the model parameters.

### Developing a nationwide representative school model for measles transmission risk

Next, we developed a nationwide school model that represents the majority of educational institutions in the U.S. The combination of the nationwide representative School Building Archetype (SBA) model and the developed multi-zone transient Wells-Riley model was used to estimate the range of measles risk that students are facing in U.S. schools. The SBA model considers two sets of parameters for primary and secondary schools investigating the impacts of students’ age and activity patterns on the risk model results. As the majority of elementary schools in the United States have self-contained classrooms [64, 65], we considered students less than 14 years old in elementary (or primary) schools with teacher self-contained educational formats and students between 14 and 18 years old in departmentalized high (or secondary) schools [66]. The estimates of quanta generation rate back-calculated from the teacher self-contained elementary and departmentalized high school case studies in the previous section were used as the *only* infection-related inputs of the SBA model. Other parameters were either assumed or culled from other references as demonstrated in Table 3. The detailed methodology for developing the SBA model is presented in Appendix C including Tables S2–S4 and Figure S2.

**Table 3:** Summary of best estimates and ranges of variables used in the nationwide representative School Building Archetype (SBA) model

Parameter	Primary School Best-Estimate [Range]	Secondary School Best-Estimate [Range]	Reference
No. of educational institutions in US 2015–2016	88,665	26,986	NCES^a^
No. of Index case/s	1	1	Assumption
Quanta generation rate (quanta / hour)	1925 [1185–3345]	2765 [1430–5140]	This Study^b^
No. of enrolled students before outbreak	513 [175–825]	854 [245–1394]	NCES^c^
Infection period in school (day)	3 [2–4]	3 [2–4]	Literature^d^
Portion of unvaccinated students	9% [8–10%]	9% [8–10%]	CDC^e^
Portion of students with ≥2-dose vaccination	91% [90–92%]	91% [90–92%]	CDC^e^
No. of students in infector’s classroom	21 [18–26]	23 [18–30]	SASS^f^
Occupancy density of classroom (m^2^/person)	4 [3–5]	4 [3–5]	DOE^g^
Occupancy density of common area (m^2^/person)	1.39 [1.04–1.74]	1.39 [1.04–1.74]	DOE^g^
Average time spent in school (mins)	400 [375–425]	400 [375–425]	SASS^h^
Average time spent in common area (mins)	20 [15–30]	30 [20–45]	NFSMI^i^
Heating and cooling periods in US schools (day)	H: 200 & C: 90	H: 200 & C: 90	Assumption^j^
HVAC system type	See Table 4	See Table 4	CBECS^k^
HVAC recirculation rate in classrooms (per hour)	6.4 [3.3–8.5]	6.4 [3.3–8.5]	Literature^l^
Outdoor air ventilation in classrooms (L/s-person)	6.7 [4.0–9.5]	6.7 [4.0–9.5]	ASHRAE^m,^
Outdoor air ventilation in common area (L/s-person)	4.9 [4.7–5.1]	4.9 [4.7–5.1]	ASHRAE^m^
HVAC runtime for applicable systems	1	1	Assumption
Air filter removal efficiency (%)	72% [44–86%]	72% [44–86%]	NAFA^n^
Infiltration rate (1/h)	0.31 [0.12–0.49]	0.31 [0.12–0.49]	DOE^g^
Deposition rate of measles bio-aerosols (1/h)	1.7 [1.0–2.7]	1.7 [1.0–2.7]	Literature^o^
Inhalation rate (m^3^/day)	12.96 [11.34–14.53]	15.53 [13.93–17.45]	EPA^p^

^a^U.S. Department of Education, National Center for Education Statistics (NCES), [66]; Table 105.50 “Number of educational institutions, by level and control of institution: Selected years, 1980–81 through 2015–16”

^b^The method explained in “Back-calculating quanta generation rate” Section and results are provided in “Estimates of quanta generation rate” Section

^c^U.S. Department of Education, NCES, [67]; Table 5 “Average student membership size of regular public elementary and secondary schools with membership, by instructional level, membership size of largest and smallest school, and state or jurisdiction: School year 2009–10”

^d^Based on existing epidemiological literature [68–70]

^e^Centers for Disease Control and Prevention (CDC) [71, 72]

^f^U.S. Department of Education, NCES, Schools and Staffing Survey (SASS) [73]; Table 7. “average class size in public primary, middle, and high schools is listed by classroom type and state in school year 2011–12”

^g^U.S. Department of Energy commercial reference building models of the national building stock, [48]; Appendix A

^h^U.S. Department of Education, NCES, SASS, [74]; “Average number of hours in the school day and average number of days in the school year for public schools, by state: 2007–08”

^i^National Food Service Management Institute [75]

^j^200 days of heating season from October to mid-April and 90 days of cooling seasons in one school academic year

^k^U.S. Energy Information Administration, Commercial Buildings Energy Consumption Survey [76, 77]

^l^Based on Polidori et al. and Chan et al. studies [78, 79]

^m^The American Society of Heating, Refrigerating and Air-Conditioning Engineers (ASHRAE) Standard 62.1–2016 Ventilation for Acceptable Indoor Air Quality (2016) [80]

^n^National Air Filtration Association [81]

^o^Based on Azimi and Stephens study [54]

^p^U.S. EPA Exposures Factors Handbook [63]

One thing to notice is, in Table 3, the best estimate values suggested for each model input could also be treated as an input model values for typical school settings. Herein, we considered six typical school settings including three primary and three secondary schools with difference types of HVAC systems and estimated the transmission risk of measles during heating and cooling seasons in those schools. The average risk of measles transmission among these six example sites was selected as our “best estimate” of measles transmission risk in US schools and shown graphs which can be found in the results section graphs.

Similar to the back-calculation process, we chose a best (primary) estimate and a range for most of SBA model variables. We applied a Monte-Carlo simulation with 10,000 iterations to account for the impacts of the model parameter ranges on the transmission risk results. Each iteration represents the risk of measles transmission in one U.S. school setup. For the Monte-Carlo simulation, we culled the model variables from two decks of primary and secondary inputs with the same proportion as the ratio of primary and secondary educational institutions in the U.S. (i.e., 76.6% of iterations were from primary school inputs and 23.3% were from secondary school inputs) [66]. A similar approach was adopted for the ratio of heating and cooling system types in the SBA model. We divided the heating and cooling systems of the U.S. schools into three categories of central-forced-air systems and ductless HVAC systems with and without air filters based on the U.S. Energy Information Administration, Commercial Buildings Energy Consumption Survey [76, 77]. The number of times that we selected each heating and cooling system type in the Monte-Carlo simulation was based on the ratio of the heating and cooling system type in the U.S. schools as summarized in Table 4.

**Table 4:** Summary of HVAC system types in U.S. schools based on the U.S. Energy Information Administration, Commercial Buildings Energy Consumption Survey

School Type	HVAC System Type	Heating	Cooling
Primary School	Central-Forced-Air	41%	26%
	Ductless with Air Filter	47%	63%
	Ductless without Air Filter	12%	10%
Secondary School	Central-Forced-Air	43%	35%
	Ductless with Air Filter	41%	54%
	Ductless without Air Filter	16%	11%

### Evaluating the effects of vaccination and airborne infection control strategies on measles transmission risk

Next, we evaluated the effects of proper vaccination (i.e., ≥2 dose vaccine, the first one after 12 months old) and various control strategies related to HVAC systems on measles transmission risk in the U.S. primary and secondary schools. To evaluate the vaccination effectiveness, we compared the measles transmission risk among unvaccinated and vaccinated cohorts in a variety of infection control scenarios in educational institution setups. To investigate the impacts of infection control strategies on the measles transmission risk, we considered three categories of infectious bio-aerosol removal approaches for schools, including improving air filter efficiencies, increasing ventilation rate, and using air purifiers in classrooms as well as their combinations. The effectiveness of the selected control strategies was evaluated by comparing the measles transmission risk after deploying the strategies with the risk in a basic-infection-control scenario of the SBA model (i.e. the removal efficiency of air filters and the ventilation rate were assumed to be equal to the minimum requirements for schools and no air purifier was used in classrooms). For each control strategy category (i.e., air filtration, ventilation, and purification), we assumed a regular and an advanced risk reduction scenario. The regular risk-reduction scenarios are costly affordable approaches compared to the advanced ones and adopted regularly for indoor environments such as schools. The advanced control scenarios are relatively extreme risk-reduction approaches and less common compared to the regular control strategies; however, they are still feasible techniques for decreasing the risk of airborne pathogens in school environments.

#### Improving removal efficiency of HVAC air filters

In the SBA model, improving the removal efficiency of HVAC air filters is limited to central-forced-air and ductless with air filter heating and cooling systems. EPA’s “Tool for School” program requires all schools to at least have air filters with MERV8 in all HVAC application [82], while the National Air Filtration Association (NAFA) recommends air filters between MERV 8 and 13 for schools [81]. On the other hand, the best commercially available air filters are called HEPA filters, and have aremoval efficiency of 99% and higher for almost all types of aerosols including droplet nuclei containing viable viral pathogens [54, 83]. Herein, for the SBA model with basic control strategies, we assumed the HVAC systems use MERV8 air filters and evaluated the changes in measles transmission risks after adopting MERV13 and HEPA filters in the heating and cooling systems as regular and advanced control scenarios, respectively.

#### Increasing ventilation rate

Many studies have highlighted the effects of outdoor air ventilation on reducing the transmission risk of measles [84–86]. ASHRAE Standard 62.1–2016 requires a default ventilation rate of 6.7 L/s-person for classrooms with students more than 9 years old changing between 4.0 and 9.5 L/s-person in various types of educational facilities [80]. It also obligates default ventilation rates of 4.7 and 5.1 L/s-person for cafeteria and dining rooms, respectively, which are considered as regular common spaces at U.S. schools in this study as explained in Appendix C [80]. For the SBA model with basic infection control designs, we assumed a minimum required ventilation rate of 6.7 L/s-person for infector’s classroom and the recirculation space, and 4.7 L/s-person in common spaces. We assumed double of the required ventilation rates in classrooms (i.e., 13.4 L/s-person) and cafeteria (i.e., 9.4 L/s-person) as the regular ventilation-related control scenario in the modeled schools. For the advanced ventilation-related control scenario, we assumed double of maximum required ventilation rate in educational facilities for the infector’s classroom and recirculation space (i.e., 19.0 L/s-person), and increased the common space ventilation rates to the double of the required ventilation rates for dining rooms (i.e., 10.2 L/s-person). Fisk (2017) summarized the reported ventilation rates in schools from several studies, where the measurements had conducted during occupancy in 20 or more classrooms [87]. The results show the maximum ventilation rate of 21.7 L/s-person in the classrooms, which demonstrates the feasibility of our advanced ventilation-related infection control scenario in schools (19.0 L/s-person in infector’s classroom and recirculation spaces), although it seems relatively excessive.

#### Using air purifiers in classrooms

Using air purifiers can reduce the transmission risk of viral airborne disease [88]. Herein, we explored the effectiveness of two air purification scenarios for reducing transmission risk of measles in the school representative model. We did not include utilizing air purifiers in classrooms for the SBA model with basic infection control design. Usually, the capacity of an air purifier for removing all particles of a given size is reported by its CADR in units of air volume per time. Although we are not aware of any standard for the size of air purifiers in indoor environments, a rule of thumb is that for every 23.2 m^2^ (250 square feet) of space about 0.0472 m^3^/s (100 cubic feet per minute - CFM) of CADR is desirable. Herein, again, we assumed regular and advanced infection control scenarios for using air purifiers *only* in the modeled schools’ classrooms as the air-purification-related control strategies. Using the rule of thumb, we assumed air purifiers with 0.189 m^3^/s (400 CFM) CADR for the modeled classrooms as the regular air purification scenario and doubled the CADR to 0.378 m^3^/s (800 CFM) in the advanced scenario.

In existing studies, several other control strategies have been deployed to reduce the transmission risk of viral airborne diseases in indoor environments including facial mask protection [89, 90], isolation [91, 92], surface disinfection [93, 94], and ultraviolet germicidal irradiation (UVGI) [44, 45, 95–98], as well as to increase the immunity of individuals by post-exposure prophylaxis after they are exposed to the infection [99]. Exploring the impacts of these control strategies was out of the scope of this study because:

(i) using facial mask is not a feasible control approach in schools particularly *before* the start of an outbreak and it is not considered as a building-related intervention
(ii) isolation strategies would be limited to closing schools in under-vaccinated communities during measles outbreaks or asking students to stay at home if they have the disease symptoms, which is not applicable for the scope of this study as herein we *only* studied the transmission risk of measles between 2 and 4 days *before* the appearance of the symptoms in the index case or start of an outbreak
(iii) we do not expect surface disinfection at the end of a school day while the students would not come back to the school at least for half a day to reduce the measles transmission risk significantly as the primary transmission pathway of measles is airborne and the measles virus usually does not survive more than a few hours outside of a human’s body
(iv) UVGI technology in schools can be deployed either by installing in-duct or by upper room UVGI air disinfection systems in the classrooms [100], while the feasibility both approaches are limited. The in-duct UVGI systems are mostly applicable to classrooms with central forced air systems, and cannot be adopted easily for every existing school (we categorized the portable air handing units with ultraviolet lights in the air purification category). Moreover, using the upper room UVGI air disinfection systems for classrooms, particularly as the primary infection control strategy *before* the outbreak started, increases the risk of overexposure to UV irradiation among students; although it is demonstrated that careful application of upper-room UVGI can be achieved without an apparent increase in the incidence of the most common side effects of accidental UV overexposure in homeless shelters [101]
(v) Post-exposure prophylaxis techniques, such as providing vaccination and immune globulin to people who are at risk for severe illness and complications from measles, are not considered as building-related interventions and increases the immunity of individuals against measles infection instead of reducing the transmission risk of measles among students

## Results

### Estimates of quanta generation rate

**Fig. 1:**
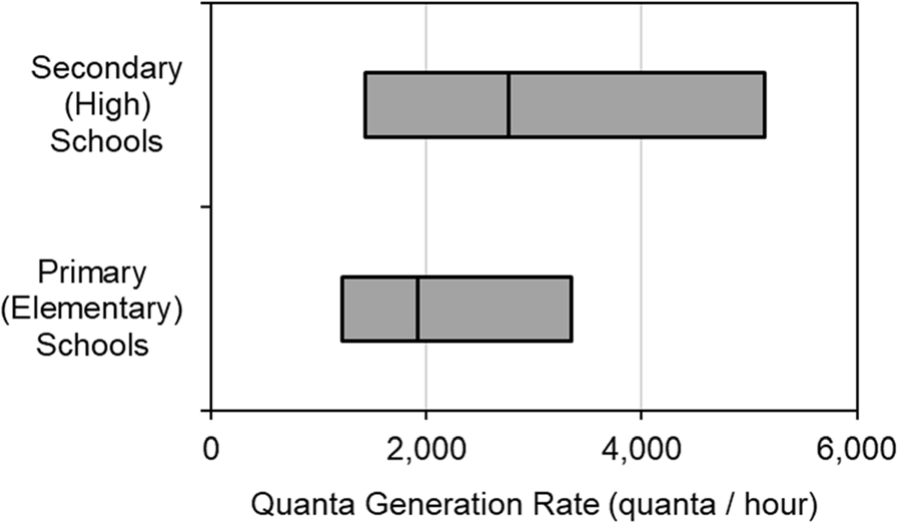
Best estimates (black line inside the boxes) and ranges of quanta generation rate for typical primary (elementary) teacher self-contained and secondary (high) departmentalized schools in the U.S.

Figure 1 demonstrates the quanta generation rate ranges and primary (best) estimates for elementary and high schools in the U.S.

Our best estimates (range) of quanta generation rate were 1925 (1185 – 3345) and 2765 (1430 – 5140), for the elementary and high school case studies, respectively. Figure 1 shows our estimates of quanta generation rate in the high school case study were significantly higher than the elementary school, which could be mostly because the high school had a departmentalized education format in which students switched between classes after each break and consequently, the index case had more interaction with the susceptible students. It is also possible that other factors, such as a longer infection period, changes in the virus infectivity in different stages of the disease (the high school index case attended her classes after rash had appeared) and types of measles virus involved in the outbreaks, caused the higher transmission rate of measles among high school students.

### Transmission risk of measles in U.S. schools

**Fig. 2:**
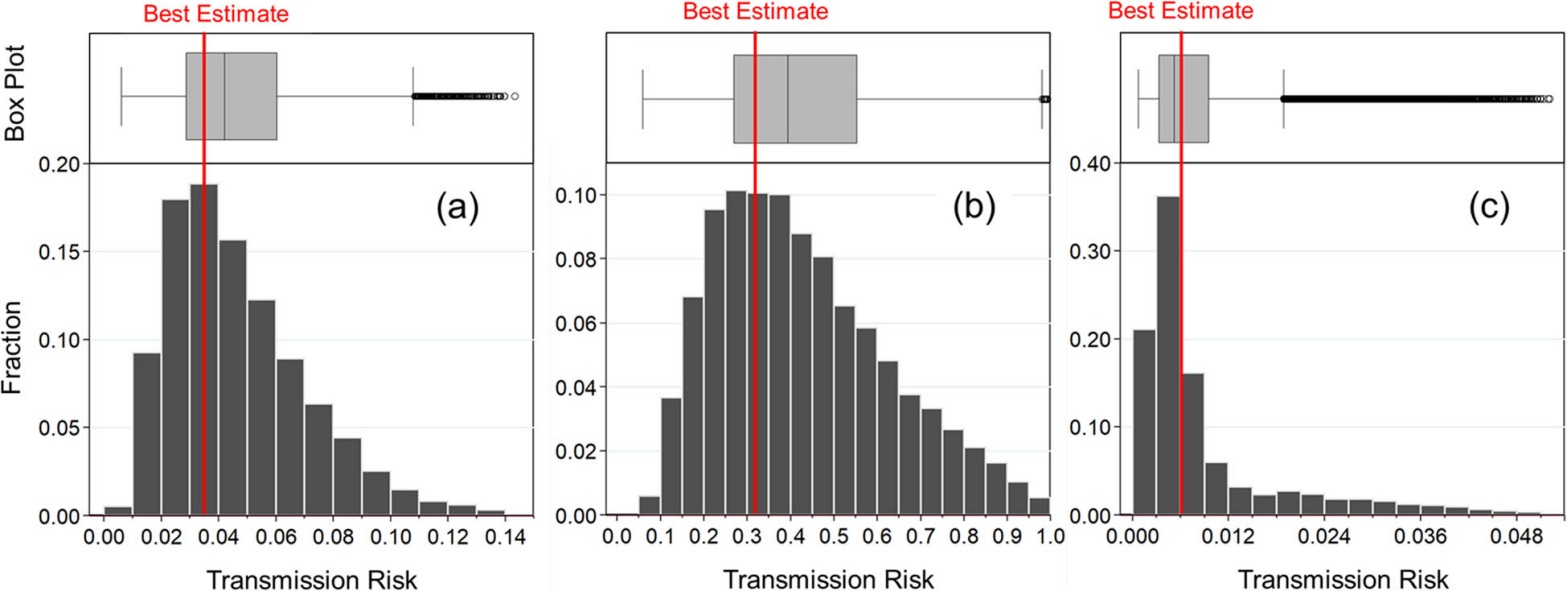
Distributions, ranges, and best estimates of measles transmission risk among (**a**) all students with an average proper vaccination coverage of 91% (changes between 90 and 92%), (**b**) unvaccinated students, and (**c**) students with proper measles vaccination assuming 1 and 5% of individuals less than 14 and between 14 and 18 years old remain susceptible, respectively

Figure 2 demonstrates the distributions, ranges and best estimates of measles transmission risk among U.S. students based on their vaccination status. The median (25th and 75th percentiles) measles transmission risk was estimated 4.2% (2.9 and 6.0%), 39.7% (27.2 and 55.6%), and 0.5% (0.3 and 1.0%) among all, susceptible, and properly vaccinated students, respectively, while our best estimates for the same outputs were 3.5, 31.9, and 0.7%, respectively. The difference between our best estimates and the median transmission risk of measles is largely associated with the difference between the mid-range and the best estimates of the SBA model variables summarizes in Table 3.

Figure 2 clearly demonstrates the importance of vaccination for reducing the risk of measles transmission in schools. Looking at the 10,000 infection transmission scenarios (iterations) in the U.S. schools, on average, the infection transmission risk among unvaccinated students is 74 times higher than properly vaccinated students with more than two doses of the vaccine. It also shows that the presence of unvaccinated students in U.S. schools increases the risk of new infection cases significantly from less than 1% if all students were vaccinated to the current nationwide best estimate transmission risk of 3.5%.

**Fig. 3:**
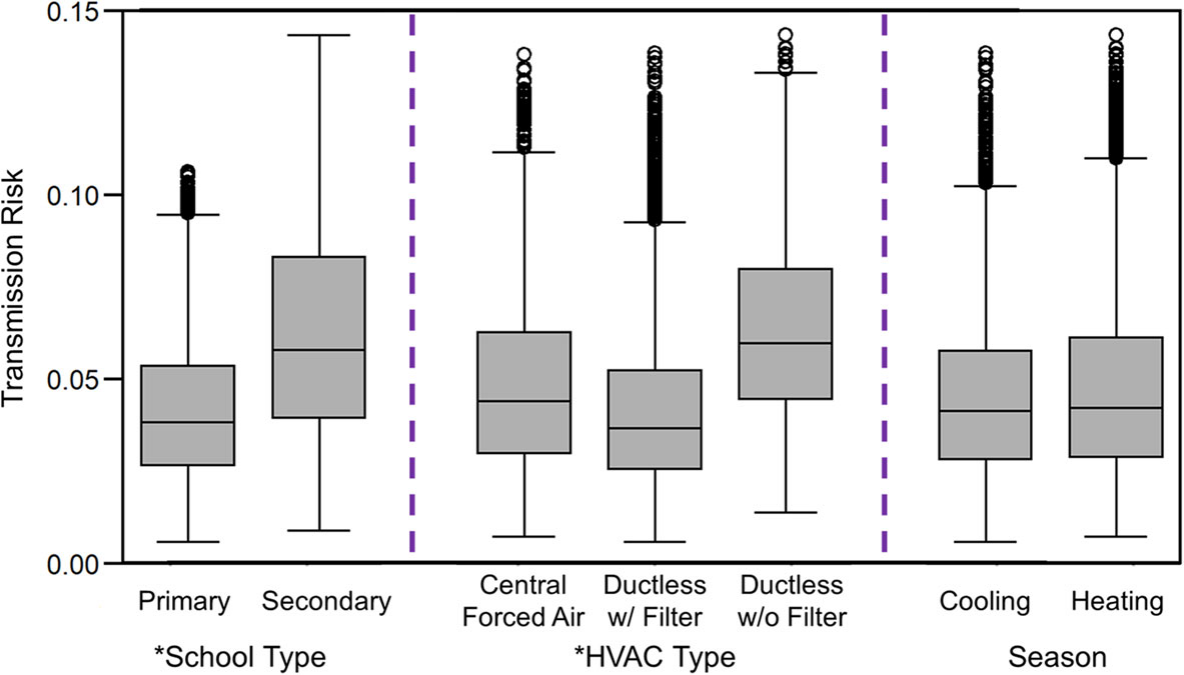
Measles transmission risk among all students in (**a**) primary teacher self-contained versus secondary departmentalized schools and (**b**) schools with central forced air and ductless with and without air filter heating and cooling systems, (**c**) schools during cooling and heating seasons. *Transmission risks were significantly different based on nonparametric Wilcoxon rank sum tests with adjusted *p*-values for the sample size (i.e., $P=1-{\left(1-0.05\right)}^{1/\sqrt{N_1{N}_2}}$, where N_1_ and N_2_ = number of iterations of compared scenarios)

Figure 3 compares measles transmission risk among all students (i.e., immunized and unvaccinated) for (i) elementary schools with teacher self-contained education systems and departmentalized high schools, (ii) three types of heating and cooling systems including central forced air and ductless with and without air filter systems, and (iii) heating and cooling seasons. The median (25th and 75th percentiles) transmission risk in elementary and high schools were 3.8% (2.7 and 5.4%) and 5.8% (3.9 and 8.3%) with the best risk estimates of 3.1 and 4.6%, respectively. The effect of HVAC system type on the measles transmission risk were lower than the education system changing the median (25th and 75th percentiles) and best risk estimates from 3.7% (2.6 and 5.2%) and 2.9% in ductless with air filter to 4.0% (2.7 and 5.7%) and 3.5% in central forced air to 6.0% (4.5 and 8.0%) and 5.5% in ductless without air filter heating and cooling systems, respectively. The effects of heating and cooling seasons on the transmission risk of measles were insignificant (using Wilcoxon rank sum test) keeping the median and best estimates of measles transmission risk during both seasons about 4.2 and 3.4%, respectively.

The best estimate of measles transmission risk was an estimated ~ 1.5 times higher in secondary departmentalized schools comparing to primary teacher self-contained ones. It is mostly because of the higher adopted quanta generation rate due to the high-interaction activity patterns of students in departmentalized schools as well as higher susceptibly rate among students between 14 and 18 years old in comparison to students less than 14 years old. The results also demonstrate using air filters in ductless-with-air-filter and central-forced-air systems decreases our best estimates of measles transmission risk ~ 47% and ~ 37% comparing to ductless-without-air-filter systems, respectively. Moreover, the lower infection transmission risk in schools with ductless-with-air-filter heating and cooling systems comparing to the ones with central-forced-air is due to the air recirculation between the infector’s classroom and other spaces. The insignificant difference between transmission risks during heating and cooling seasons demonstrates the variation in types of HVAC system used for heating and cooling in schools (Table 4) does not influence the model results significantly.

**Fig. 4:**
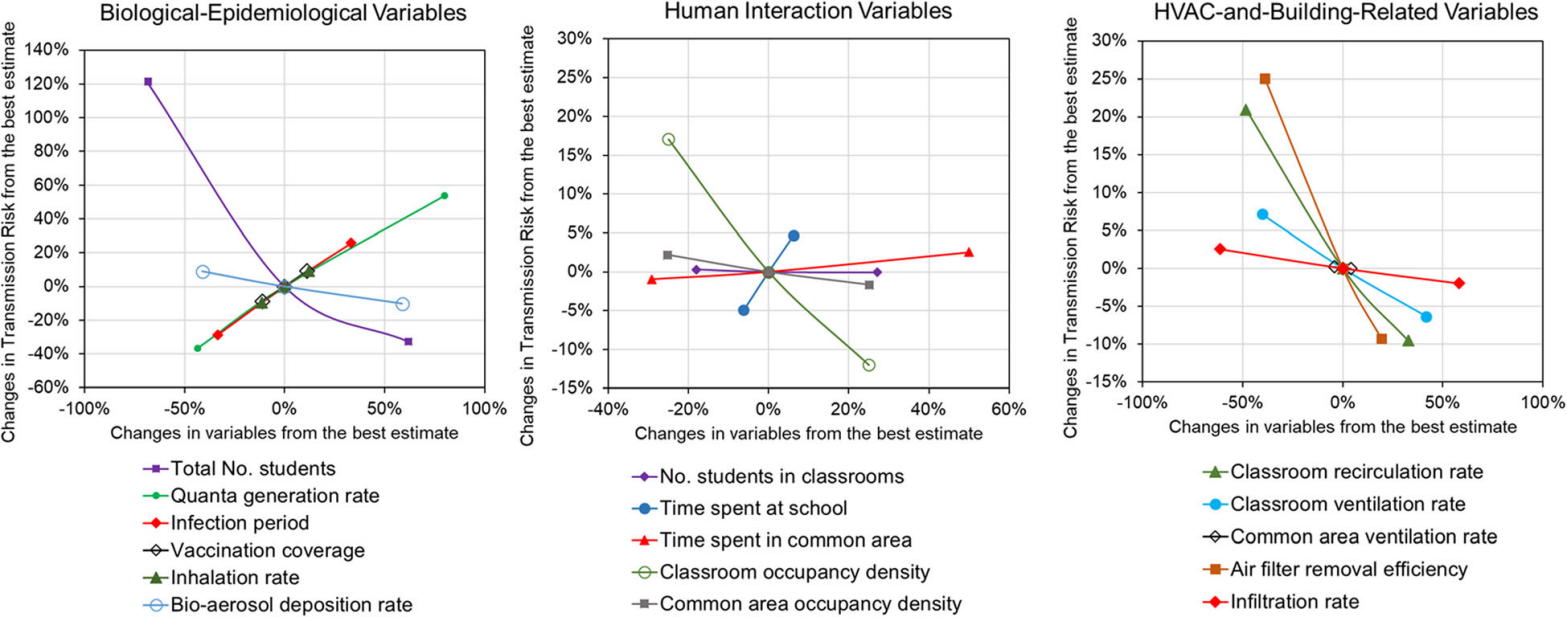
Sensitivity of the measles transmission model in U.S. schools to changes in (**a**) biological-epidemiological variables, (**b**) human-interaction-related parameters and (**c**) HVAC-building-related variables

Next, we explored the sensitivity of the results to the changes in the SBA model variables. We divided the SBA model variables into three categories of biological-epidemiological, human interaction, and HVAC-and-building-related variables, as demonstrated in Fig. 4. In each diagram, the x- and y-axis values show the changes in the model variables and the transmission risks associated with those changes, respectively. In the diagrams, each branch shows the relative variation of a model parameter from its primary estimate and the associated relative changes in the transmission risk estimates. The lengths of branches were limited to the ranges of the SBA model variables demonstrated in Table 3. The sensitivity analysis approach is described in detail in Appendix D including Figure S3 and Tables S5–6.

Figure 4 results show the higher impacts of biological-epidemiological variables on the measles transmission risk estimates compared to the other two variable categories. On average, the biological-epidemiological, HVAC-and-building-related, and human interaction variables alter the estimates of measles transmission risk 59, 17, and 9% compared to our best infection risk prediction, respectively. Among the biological-epidemiological variables, the model outcomes are most sensitive to the range of number of enrolled students. This drastic change in the transmission risk associated with number of enrolled students is actually driven by the variation in the ratio of infected cases in the infector’s classroom versus the number of enrolled students. Next influential variable is quanta generation rate which changes the transmission risk from 37% less to 54% higher than the primary transmission risk estimate when the lowest and highest estimates of the variable are adopted in the SBA model. The range of infection period is the third most impactful model variable among all parameters changing the estimates of infection risk from 29% less to 26% higher than the primary risk estimation. The occupancy density of classrooms has the highest impact on the measles infection risk among human interaction variables varying the risk from 12% lower to 17% higher risks in comparison to the primary risk estimates when the high and low variable estimates are used in the model, respectively. In the HVAC-and-building-related category, removal efficiency of air filters and recirculation rate of classrooms are the two high impactful variables, shifting the transmission risk from 9 and 10% lower to 25 and 21% higher risk estimates comparing to the primary transmission risk scenario, respectively.

### Effects of selected infection control strategies on measles transmission

**Fig. 5:**
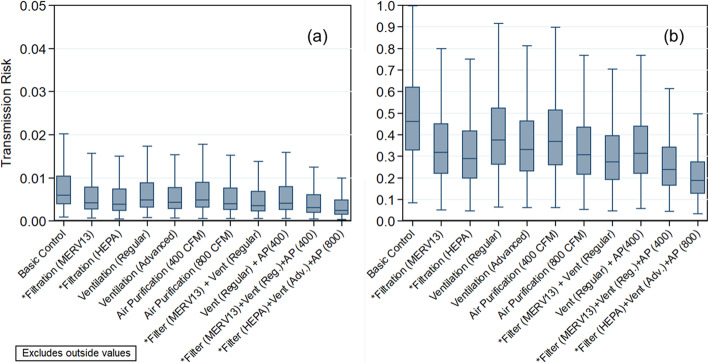
Transmission risk of measles among (**a**) properly immunized and (**b**) unvaccinated students and the effects of 10 infection control strategies including regular and advanced filtration, ventilation, and air purification (AP) techniques and their combinations

Next, we explored the effects of vaccination, three categories of common infection control strategies suitable for school environments (i.e., enhancing air filtration, ventilation, and purification) and their combinations on transmission risk of the measles virus. For each infection control category, we evaluated the effectiveness of two scenarios including one regular and one advanced infection removal approach. Moreover, we investigated the infection removal effectiveness of four combination scenarios including (i) regular filtration and ventilation, (ii) regular purification and ventilation, (iii) regular filtration, ventilation, and purification, and (iv) advanced filtration, ventilation, and purification improvements in the infection control designs of the SBA model. It is noticeable that the filtration scenarios were only applied to central-forced-air and ductless with air filter systems in the SBA model. Figure 5 compares the transmission risk of measles for various infection control strategies.

The first and most important implication from Fig. 5 is the importance of vaccination. The results show regardless of the adopted control strategy, the risk of infection transmission among unvaccinated students is significantly higher than the immunized students. It is shown while the median transmission risk of measles among properly immunized students in schools with basic control strategy is estimated 0.6% and for all other control scenarios remains below 0.5%, the median infection risk among unvaccinated students ranges between 46 and 20% for the basic control designs and the combinations of advanced infection removal strategies, respectively. This demonstrates even with the most advanced infection control mechanisms the measles transmission risk among unvaccinated cohorts remains more than 30 times higher than the risk among properly immunized students staying in school environments with basic infection control designs.

Figure 5-b highlights the role of building designs and particularly effects of adopting regular and advanced control strategies and their combinations on reducing the transmission risk of measles in schools among unvaccinated (susceptible) cohorts. Considering central-forced-air and ductless-with-air-filter heating and cooling systems only, upgrading HVAC air filters from MERV8 in the basic control scenario reduced the median (1st and 3rd quartiles) of infection risk among unvaccinated students from 45% (32 and 60%) to 32% (22 and 45%) and 29% (20 and 42%) using MERV13 and HEPA filters, respectively. Enhancing ventilation rates decreased the median (1st and 3rd quartiles) infection risk from 46% (33 and 62%) among unvaccinated students in the basic control scenario (considering all types of HVAC systems) to 38% (26 and 52%) and 33% (23 and 46%) in the regular and advanced infection control scenarios, respectively. It is also shown that deploying air purifiers in classrooms could be more efficient than the explored ventilation scenarios, which reduced the median (1st and 3rd quartiles) infection risk to 37% (26 and 51%) and 31% (22 and 44%) for air purifiers with CADR of 400 CFM and 800 CFM, respectively.

Figure 5 also demonstrates the effects of adopting more than one control strategy at a time on the transmission risk of measles. Deploying two regular control approaches reduced the median (1st and 3rd quartiles) of measles transmission risks among susceptible students to 28% (19 and 40%) and 31% (22 and 44%) when combinations of regular improvements in filtration-ventilation and ventilation-purification approaches are used in the applicable school environments, respectively. Applying filtration, ventilation, and purification techniques together lowered the median (1st and 3rd quartiles) of measles transmission risk to 24% (16 and 34%) and 19% (13 and 28%) for regular and advanced infection control strategies, respectively.

**Fig. 6:**
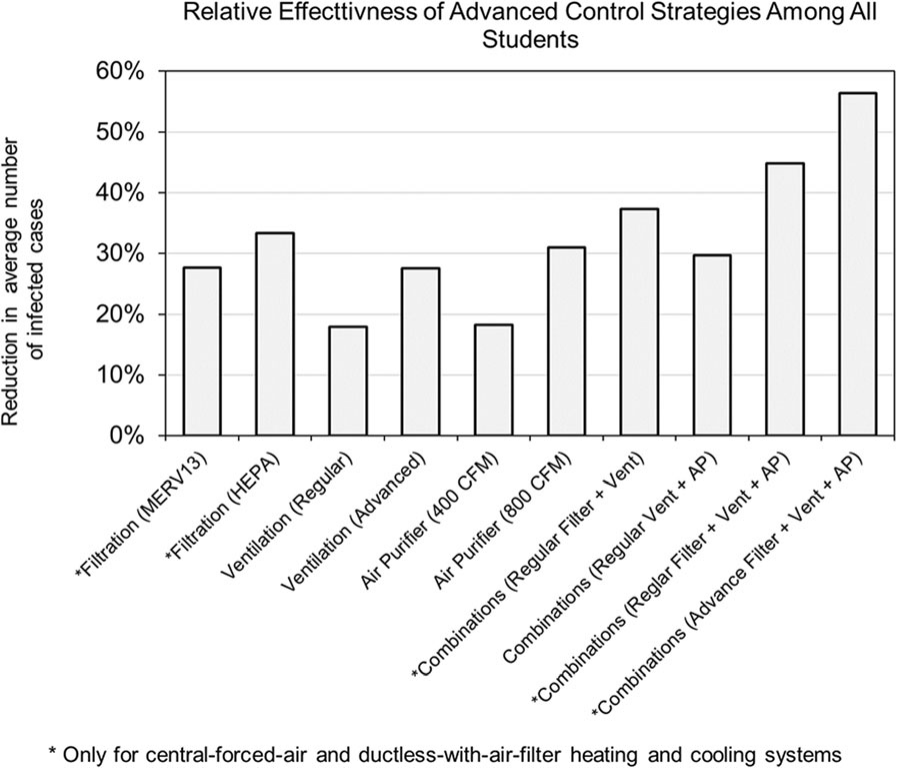
Relative effectiveness of advanced control strategies on measles transmission risk among all students

Figure 6 compares the relative effectiveness of the adopted control strategies, which were estimated by comparing the average number of infected cases among all students in the basic infection control designs with the same numbers after enhancing the removal rates of infectious bio-aerosols in the SBA model using different control scenarios. For the control scenarios related to improving the effectiveness of air filters, we only considered the central-forced-air and ductless-with-air-filter heating and cooling systems.

Figure 6 results show for the modeled heating and cooling systems, upgrading the air filters to MERV13 and HEPA filters reduce the average number of infected students by approximately 28 and 33%, respectively. Ventilation-related control strategies had average effectiveness of 18 and 28% for regular and advanced scenarios, respectively. Placing air purifiers with regular CADR of 400 cfm in typical school classrooms decreased the number of infected cases by 18%, while doubling the CADR of the air purifiers in the advanced control scenario to 800 cfm increased the effectiveness of the control method to 31%. Using two regular infection control scenarios increased the effectiveness of the control strategies to 37 and 30% when combinations of filtration-ventilation and ventilation-purification scenarios were adopted, respectively, showing the combinations of two regular control approaches can be as effective as adopting one advanced control strategy. Combining all regular and advanced control scenarios reduced the average number of infected cases up to 45 and 56%, respectively, demonstrating the potentially high impacts of building designs on avoiding the new cases of airborne disease infections in school setups.

## Discussion

We used a combination of a newly developed multi-zone Wells-Riley approach, a nationwide representative School Building Archetype (SBA) model, and a Monte-Carlo simulation to estimate the transmission risk of measles among U.S. students. In the multi-zone Wells-Riley model, we considered several microenvironments within a typical school building (i.e. infector’s classroom, recirculation space, and common space) and simulated the transmission of measles virus between the zones based on the building and epidemiological characteristics of schools adopted from the SBA model. In the SBA model, we considered two education formats (i.e., teacher self-contained and departmentalized schools) for U.S. schools affecting the interaction of susceptible students with index cases, three categories of HVAC systems (i.e., central-forced-air and ductless with and without air filter systems), and changes in susceptibility of students based on their age and vaccination status. Finally, we investigated the effectiveness of vaccination and ten control strategy scenarios related to enhancing air filtration, ventilation, and purification rates in schools for reducing the risk of measles transmission among primary and secondary students.

As expected, vaccination was shown to be the primary approach for reducing the transmission risk of measles among students. However, the risk still exists when a contagious kid in school encounters vaccinated individuals; therefore, further motivation for our work was understanding the role of factors that influence disease transmission beyond vaccination. Here, we found school educational formats and building and HVAC characteristics play critical roles in measles transmission. Specifically, we found that the transmission risk of measles in primary schools (assuming teacher self-contained classrooms) is less than secondary schools (assuming departmentalized systems) and schools with ductless-with-air-filter and ductless-without-air-filter systems have the lowest and highest transmission risks of measles, respectively.

### Comparing the risk model assumptions and results with existing epidemiological studies

In this section, we evaluated our assumptions for the susceptibility of students to measles virus based on their age and vaccination coverage and our best estimates of measles transmission risk among susceptible students as the primary outcome of this study.

We assumed students less than 14 years old are 100, 10 and 1%, susceptible to measles if they were not vaccinated, had one dose of measles vaccine, and had two doses of the vaccine, respectively, and students between 14 and 18 years old are 100 and 5% susceptible to measles viruses if they were not vaccinated and had either one or two doses of the vaccine, respectively. In Tables 5 and 6, we compared our assumptions for susceptibility of students with the reported number of infected cases and measles attack rates during 27 measles outbreaks in primary and secondary schools in developed countries. The selected studies provided information on the total number of enrolled students during the outbreak, final number of infected cases, and vaccination coverage of the students. In all cases, one index case started the outbreak and infected at least one other susceptible student at their school. During the outbreaks if a portion of students were vaccinated or revaccinated, we considered the final vaccination coverage of students in the analysis. We also culled information regarding the number of received vaccination doses, attack rates among unvaccinated (ARU) and immunized (ARI) individuals, and number of infected cases in the first generation of the outbreaks when the data were provided. For validation purposes, we expect the reported measles attack rates among immunized individuals with one dose of vaccination (ARI-1-Dose) and two doses of vaccination (ARI-2-Dose) remains lower than the assumed susceptibility rates for the cohorts and the estimated number of susceptible individuals to be larger than the final number of infected cases during the outbreaks.

**Table 5:** Characteristics of measles outbreaks in primary schools

School Location [outbreak year] – Type	NO. Students	No Vac. Record	1-Dose Vaccination	2-Dose Vaccination	ARU	ARI-1- Dose	ARI-2- Dose	ARI-General	Susceptible Students	NO. First Gen. Cases	Total NO. Cases
New York, US [1945–46] ES I [44]	367	100.0%	0.0%	0.0%	77.6%	N/A	N/A	N/A	170	26	132
New York, US [1945–46] ES II [44]	530	100.0%	0.0%	0.0%	83.9%	N/A	N/A	N/A	249	64	209
New York, US [1945–46] ES III [44]	492	100.0%	0.0%	0.0%	69.4%	N/A	N/A	N/A	193	123	134
Kansas, US [1970] ES [102]	690	14.2%	NR	NR	30.3%	NR	NR	2.6%	122	3	35
New York, US [1974] ES [33]	868	3.3%	NR	NR	20.7%	NR	NR	6.4%	113	28	60
Texas, US [1985] JHS I [103]	1141	0.6%	87.6%	11.9%	10.0%	4.5%	**1.4%**^a^	4.1%	107	10	21
Texas, US [1985] JHS II [103]	1122	1%	NR	NR	NR	NR	NR	NR	122	NR	34
East Sussex, UK [1992–93] E-M-HS [104]	1673	31.5%	NR	NR	17.8%	NR	NR	1.52%	528	41	66
Alaska, US [1996] MS [61, 105]	687	< 1%	45%	44%	NR	NR	NR	< 2.18%	41	4	15
Alaska, US [1996] ES [61, 105]	525	< 1%	45%	44%	NR	NR	NR	< 1.33%	31	4	7
Reuler, Luxembourg [1996] PS [106]	363	22.8%	NR	NR	54.7%	NR	NR	4.6%	102	28	45
Wincrage, Luxembourg [1996] PS [106]	343	28.0%	NR	NR	51.9%	NR	NR	1.0%	110	15	43
Disburg City Germany [2006] E-M-HS [107]	1250	3.8%	24.5%	58.4%	52.8%	1.0%	0.4%	0.5%	81	NR	53
California, US [2008] ES [108]	377	10.9%	NR (< 50%)	NR (> 50%)	9.8%	0.0%	0.0%	0.0%	62	2	4
Beijing, China [2014] ES [109]	1245	0.5%	1.7%	97.8%	0.0%	0.0%	0.9%	0.9%	20	3	11

*PS* Primary School; *ES* Elementary School; *MS* Middle School; *JHS* Junior High School; *E-M-HS* Elementary, Middle, and High School combined; *N/A* Not applicable; *NR* Not Reported.

^a^ Only reported outbreak where ARI-2-Dose was larger than assumed measles susceptibility rate of students less than 14 years old with 2-dose of vaccination (i.e. 1%)

**Table 6:** Characteristics of measles outbreaks in secondary schools

School Location [outbreak year] – Type	NO. Students	No Vac. Record	1-Dose Vaccination	2-Dose Vaccination	ARU	ARI-1- Dose	ARI-2- Dose	ARI, General	Susceptible Students	NO. First Gen. Cases	Total NO. Cases
Massachusetts, US [1984] SHS [59]	2098	0.6%	42.3%	57.1%	22.9%	1.7%	0.5%	1.0%	117	5	24
Texas, US [1985] HS [103]	1796	0.6%	87.6%	11.9%	10.0%	4.5%	1.4%	4.1%	99	3	5
Illinois, US [1985] HS [22]	1873	0.3%	71.1%	28.9%	0.0%	4.5%	1.7%	3.7%	100	69	69
New Mexico, US [1987] HS [110]	2012	1.9%	76.5%	21.6%	0.0%	2.8%	1.7%	2.6%	137	24	49
Texas, US [1989] HS [111]	2243	0.0%	95.2%	4.8%	N/A	3.2%	1.9%	3.2%	112	58	71
Honkajoki, Finland [1989] HS [112]	144	63.8%	29.5%	6.7%	22.4%	**29.0%**^a^	**14.3%**^a^	**26.3%**^a^	69	22	25
Gwynedd, North Wales [1991] SHS [113]	723	38.5%	NR	NR	33.1%	NR	NR	1.0%	140	12	45
Alaska, US [1996] HS [61, 105]	127	< 1%	45.0%	44.0%	NR	NR	NR	< 3.94%	7	3	5
Alaska, US [1996] HS I [114]	2192	0.0%	48.8%	50.4%	0.0%	1.5%	0.0%	0.7%	110	16	17
Alaska, US [1996] HS II [114]	1486	0.5%	53.9%	45.5%	0.0%	0.1%	0.3%	0.1%	81	NR	2
Pennsylvania, US [2003] SBS [115]	663	0.5%	3.9%	94.9%	66.7%	0.0%	1.0%	0.9%	36	5	8
Quebec, Canada [2011] HS [116]	1306	4.7%	9.0%	85.5%	82.0%	4.6%	3.7%	4.7%	123	10	110

*HS* High School; *SHS* Senior High School; *SBS* Senior Boarding School; *NR* Not ffig Reported.

^a^ Only extreme measles outbreak where ARIs were larger than assumed measles susceptibility rate of students between 14 and 18 years old with either 1-dose or 2-dose vaccination (i.e. 5%)

Table 5 summarizes the characteristics of 10 measles outbreaks in the U.S. and 5 outbreaks in other developed countries in primary schools with the average age of students less than 14 years old. Seven of the summarized studies in Table 5 did not provide information about the portion of students who had received one or two doses of measles vaccine. In these cases, we assumed the immunized students had received one dose of the measles vaccine. In all cases, the number of estimated susceptible individuals was higher than the final number of infected cases at the end of the outbreaks.

The maximum ARU in primary schools listed in Table 5 was 84% during an outbreak in New York State, between 1945 and 1946, although the school had deployed a UVGI control system to prevent the spread of measles. The high attack rate of measles during this outbreak and the other two outbreaks in New York State reported in the study by Perkins et al. [44] shows the validity of our assumption for 100% susceptibility to measles among unvaccinated cohorts. The maximum ARI was 6.4% reported in the study by Riley et al. [33], where the ARI-1-Dose and ARI-2-Dose values were not mentioned. Considering the fact that the outbreak happened in 1974 when 2-dose vaccination was not common and assuming immunized students, in this case, had received one dose of vaccination, the maximum ARI in primary schools was in line with our 10% assumption of susceptibility to measles among students under 14 years old who only receive a 1-dose vaccine. The ARI-2-Dose values summarized in Table 5 were also lower than our assumption of 1% susceptibility between primary school students except for one Texas junior high school case, where the attack rate was 1.4%.

Table 6 demonstrates the characteristics of 12 measles outbreaks (9 in the U.S. and 3 in other countries) in secondary schools with the average age of students between 14 and 18 years old. Most of the summarized articles (10 out of 12 studies) reported the proportion of students who had received one or two doses of measles vaccine as well as the measles attack rate during the studied outbreaks. The measles attack rate in all cases remained lower than our assumption of the vaccine failure among students between 14 and 18 years old (i.e. 5%) except in one extreme outbreak in Finland where 29 and 14.3% of students who had received one dose and two doses of measles vaccine, respectively, were infected. It is noticeable that five of the ARI-2-Dose values were between 1 and 5%, demonstrating our assumption for higher vaccination failure among students between 14 and 18 years old receiving two doses of measles vaccine comparing to students less than 14 years old was valid.

We also estimated the number of avoided infected cases by reducing the total number of infected cases from the number of susceptible students. The results show that other control mechanisms than the vaccination such as air filtration, ventilation, and purification and isolation of suspicious infected cases during the outbreaks, on average, could protect ~ 60% (ranged between 11 and 98%) of susceptible students from the infection in the studied outbreaks. This demonstrates the importance of paying attention to other control strategies in addition to the vaccination to reduce the transmission risk of measles in the built environment.

**Fig. 7:**
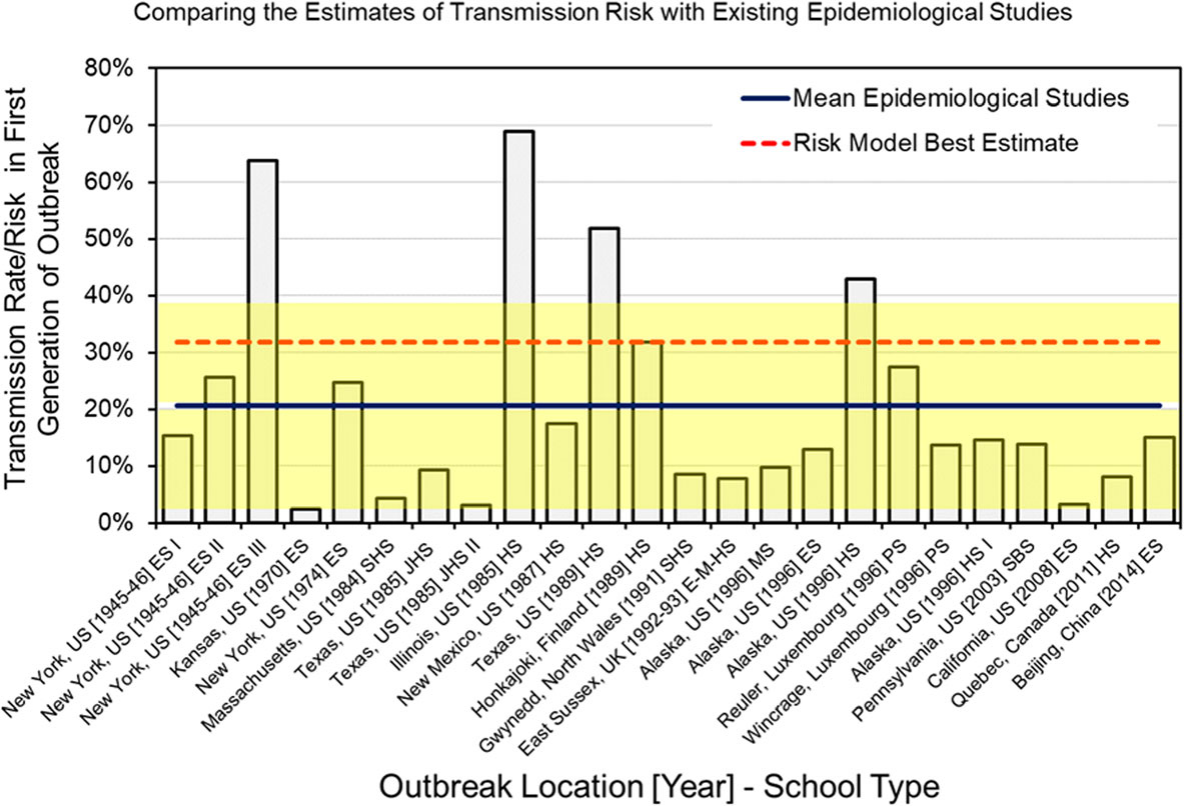
Comparing our best estimate of nationwide measles transmission risk among susceptible students in U.S. schools with estimated transmission rates of measles during first generations of the infection outbreaks in developed countries’ schools among susceptible students reported in existing epidemiological studies

The primary outcome of the developed Wells-Riley model is the infection risk defined as the number of infected cases divided by the number of susceptible individuals during one generation of the infection outbreak as demonstrated in Eq. 1. Therefore, to evaluate our developed model results, we compared our best estimate of nationwide infection risk of measles in U.S. schools with the estimated transmission rate of measles during the first generation of the infection outbreaks in schools in developed countriesreported in existing epidemiological studies. Totally, the number of infected cases during the first generation of measles outbreaks was reported in 24 summarized studies in Tables 5 and 6. The measles transmission rates were estimated by dividing the reported number of infected cases during the first generation of the outbreaks to the estimated number of susceptible students in each school as shown in Fig. 7.

Most of the demonstrated studies in Fig. 7 did not directly report the number of infected cases during the first generation of the outbreaks; instead, they reported the timeline when the infected cases were detected. In these cases, we considered a 14-day (± 7 days) incubation period (i.e., the time elapsed between exposure to a pathogenic organism, and when symptoms and signs are first apparent) for measles, which means we considered the infected cases in a 7-to-21 time period after the index cases had entered the schools as individuals who were infected during the first wave of the outbreaks.

Figure 7 shows an average (± SD) first-generation measles transmission rate of 21% (±18%) for the reported outbreaks in developed countries’ primary and secondary schools, ranging between 3 and 69%, while our best nationwide transmission risk estimate is 32% among susceptible students. We believe the higher estimate of transmission risk derived from the developed Wells-Riley model does not necessarily mean that the developed model overestimates the infection risks as long as our risk estimates are within the range (i.e., average ± SD) of reported first-generation transmission rates, because:

(i) The number of selected outbreaks are limited and they are not representative of U.S. schools
(ii) Except the two studies used for the quanta generation rate back-calculation process, other selected studies have not provided enough information regarding the student activities, school building properties, and epidemiological characteristics of the outbreaks; therefore, we are unaware of the infection control strategies deployed in most of these schools during the outbreaks

For these reasons, the fact that the primary outcome (i.e. transmission risk among susceptible students) of our risk model is within the range (average ± SD) of the reported measles transmission rates found in the existing literature demonstrates the validity of our models adopted in this study.

We also demonstrated the estimated transmission risk of measles in six typical US school settings among susceptible students (based on our best estimates of school building characteristics presented in Table 3) in Appendix E (Figure S4) and compared them with estimated transmission rates of measles during the first generations of the infection outbreaks in schools from developed countries among susceptible students reported in existing epidemiological studies.

### Implications

The results of the current simulation study indicate the primary importance of vaccination for reducing the risk of measles transmission among students at schools. Additionally, our results related to the estimated distribution and range of measles transmission risk in school can aid epidemiologists and risk analyzers to evaluate the chance of a new outbreak in a community. Moreover, the study outcomes shown in Figs. 5 and 6 clearly demonstrate that beyond vaccination, several factors such as increasing filtration, ventilation and air purification rates in indoor environments also were influential in disease transmission. However, none of these interventions were as effective as vaccination and should not be used as a basis or control strategy in place of vaccination. Our findings support their use as a supplemental control strategy that must be combined with vaccination.

In this study, for the first time, we developed a nationwide representative School Building Archetype (SBA) model and a transient multi-zone Wells-Riley model for estimating the transmission risk of an airborne infectious agent (i.e., measles viruses) among U.S. students. We also demonstrated that the combination of the SBA and transient multi-zone Wells-Riley models estimates the nationwide infection risk of measles within the range (i.e., average ± SD) of first-generation transmission rates of measles in schools according to the existing epidemiological studies (Fig. 7). As the only three biological-related variable were involved with the SBA and Wells-Riley models (i.e., quanta generation rate, infection period, and deposition rate), the newly developed models are also capable of estimating the nationwide transmission risk of other airborne infectious disease in school environments as long as the biological-related variables of the desired airborne pathogen are available. Moreover, the methods used in this study can be expanded to other indoor environments such as offices, healthcare facilities, and residences. Therefore, policy makers and standard developers can adopt the methodology used in this study to establish new nationwide and regional policies and requirements for school or other indoor environments to reduce the chance of spread of infectious airborne disease.

We also evaluated the impacts of HVAC system designs, educational format of schools, and several control strategies on the transmission risk of measles in schools in Figs. 3 and ***5*** and compared the effectiveness of a variety of infection control approaches on reducing the average number of infected cases in the SBA model in Fig. 6. Expanding the framework of this study to other airborne infectious diseases, would provide additional information for building designers and decision-makers to consider before selecting the most appropriate HVAC system types for school buildings. Moreover, the method used in this study is not limited to nationwide estimates of the airborne infection transmission risks, but it can also be deployed by building designers to predict the transmission risk of a variety of infectious diseases in a specific school environment during the design process. For example, as demonstrated in Fig. 3, the transmission risk of measles in schools with ductless HVAC systems and a proper air filter is lower than the other two types of HVAC systems, which most probably would be the same condition for other airborne infectious diseases. As another implication, the comparison between the effectiveness of various control strategies will help school officials to select a financially appropriate infection control approach based on the school’s building and HVAC system characteristics to reduce the transmission risk of measles or other infectious airborne diseases in an existing school building. For example, results shown in Fig. 6 demonstrate adopting HEPA filters instead of MERV13 in HVAC systems would improve the filtration effectiveness less than 5%, while existing studies estimated annual costs of HEPA filters are more than double of MERV13 air filters [54] or if advanced infection control strategies cannot be deployed for a school because of high installation costs or building and HVAC system properties, adopting a combination of two regular infection control scenarios can provide a similar or even higher removal rates of infectious bio-aerosols.

Herein, for the first time we back-calculated quanta generation rate for one infectious disease from multiple studies. This approach helped us to capture a wider potential range for measles quanta generation rate considering different school setups (Fig. 1), which canalso be adopted by researchers in future studies to back-calculate the quanta generation rate of other airborne pathogens. Moreover, the sensitivity analysis (Fig. 4) determined what model variables have the highest impacts on the measles transmission risk estimates in schools. The outcomes of the sensitivity analysis help researchers to identify the critical parameters in the risk model and highlights the most influential research pathways for future studies.

### Limitations

One of the most challenging parts of this simulation effort was to find proper ranges for the SBA model variables representing the majority of school building stock in the U.S. The variable ranges were particularly essential for parameters that have high impacts on the risk results including quanta generation rate, infection period, density of students in infector’s classroom, and classrooms’ recirculation and filtration rates. However, the existing knowledge around the ranges of many of the model variables is limited, particularly for quanta generation rate and air recirculation rate in schools. For the quanta generation rate, we only found two studies describing the characteristics of measles outbreaks in schools from 1974 and 1989 [22, 33] in such details that could be used in the back-calculation process; and for recirculation rate, we relied on one study [78] measuring the average air recirculation rate in nine California classrooms. Although the study by Riley et al. is the most well known article used for estimating the range of quanta generation rate, and measurments in the study byPolidori et al. have been used as the representative of U.S. classrooms’ recirculation rate in other peer-reviewed articles [79], more comprehensive studies are required for evaluating the ranges of these variables.

The Wells-Riley approach only considers the airborne transmission pathways of infectious diseases while, the transmission risk of measles through other pathways including fomite and direct contact remains unclear. Although, it is shown that measles primary transmission pathway is airborne, it is necessary to perform more research on the other pathways of measles transmission. This limitation would be more critical for other airborne infectious diseases such as influenza and coronavirus which fomite and direct contact are also shown to have a considerable influence on the risk results.

In developing the transient Wells-Riley model, we made several simplifications such as assuming continuous stay of students in the microenvironments, constant number of students, and a simplified format of student interactions. Although these factors were considered in some levels during the back-calculation process, for individual case studies (not nationwide simulations), where more information on building and human interaction characteristics is available, more advanced and complex derivations of the Wells-Riley approach or other mathematical and statistical models should be deployed.

## Conclusion

We used a combination of a newly developed transient multi-zone Wells-Riley approach, a nationwide representative School Building Archetype (SBA) model, and a Monte-Carlo simulation to estimate the transmission risk of measles among students in U.S. schools. We also estimated the number of susceptible students for a school setup based on the age and vaccination record of students and back-calculated quanta generation rate of measles for our newly developed risk model based on two existing epidemiological studies. We considered three microenvironments within school buildings, two education formats, and three types of HVAC systems in the risk model and used the Monte-Carlo simulation with 10,000 iterations to examine the effects of model parameter ranges on the risk results. Our best estimates of nationwide transmission risk of measles in U.S. school were 3.5 and 32% among all and susceptible students, respectively. The results of our study show the transmission risk of measles among unvaccinated students is more than 70 times higher than properly immunized ones. In the back-calculation process, we estimated the quanta generation rate of 2765 and 1925 quanta per hour for primary schools with teacher self-contained classrooms and secondary schools with departmentalized system, respectively, showing that higher student interactions in the departmentalized schools significantly increases the transmission risk of measles. Comparing various types of HVAC systems shows schools with ductless-with-air-filter systems have the lowest transmission risk of measles, while the risk is highest for schools with ductless-without-air-filter systems. Finally, exploring the effectiveness of 10 control scenarios for reducing the transmission risk of measles in schools shows a large difference among the effectiveness of various control strategies and the selected infection control approaches can reduce the average number of infected cases up to 56% when a combination of advanced air filtration, ventilation, and purification approaches was adopted.

## Supplementary information

**Additional file 1.**

## Data Availability

The datasets used and/or analyzed during the current study are available from the corresponding author on reasonable request.

## Abbreviations

AP: Air Purifier
ARI: Attack Rates among Immunized
ARI-1-Dose: Attack Rates among Immunized individuals with one Dose of vaccination
ARI-2-Dose: Attack Rates among Immunized individuals with two Dose of vaccination
ARU: Attack Rates among Unvaccinated individuals
ASHRAE: American Society of Heating, Refrigerating and Air-Conditioning Engineers
CADR: Clean Air Delivery Rate
CDC: Centers for Disease Control
CFD: Computational Fluid Dynamics
CFM: Cubic Feet per Minute
DOE: Department of Energy
E-M-HS: Elementary, Middle, and High School
EPA: Environmental Protection Agency
ES: Elementary School
HEPA: High-Efficiency Particulate Air
HS: High School
HVAC: Heating, ventilation, and air-conditioning
ID63: 63% Infectious Dose
IgG: Immunoglobulin G
JHS: Junior High School
MERV: Minimum Efficiency Reporting Value
MS: Middle School
NAFA: National Air Filtration Association
NCES: National Center for Education Statistics
NR: Not Reported
PS: Primary School
SASS: Schools and Staffing Survey
SBA: School Building Archetype
SBS: Senior Boarding School
SD: Standard Deviation
SHS: Senior High School
SIR: Susceptible-Infectious-Recovered
SIS: Susceptible-Infector-Susceptible
UVGI: Ultraviolet Germicidal Irradiation
