# Correction to: Estimating the nationwide transmission risk of measles in US schools and impacts of vaccination and supplemental infection control strategies

**DOI:** 10.1186/s12879-020-05259-1

**Published:** 2020-07-24

**Authors:** Parham Azimi, Zahra Keshavarz, Jose Guillermo Cedeno Laurent, Joseph G. Allen

**Affiliations:** Department of Environmental Health, Harvard T. H. Chan School of Public Health, Boston, USA

**Correction to: BMC Infect Dis (2020) 20:497**

**https://doi.org/10.1186/s12879-020-05200-6**

Following publication of the original article [1], the authors identified an error in the author name of dr. Jose Guillermo Cedeno Laurent.

The incorrect author name is: Jose Guillermo Cedeno Cedeno Laurent.

The correct author name is: Jose Guillermo Cedeno Laurent.

The author group has been updated above and the original article [1] has been corrected.
