# Supplementary material for: Estimating the nationwide transmission risk of measles in US schools and impacts of vaccination and supplemental infection control strategies
Source: BMC Infect Dis. 2020 Jul 11;20:497. doi: 10.1186/s12879-020-05200-6 (PMC7351650; doi:10.1186/s12879-020-05200-6)
Supplement: Supplementary file 1 — Additional file 1. [file 12879_2020_5200_MOESM1_ESM.docx]

**Estimating the Nationwide Transmission Risk of Measles in US Schools and Impacts of Vaccination and Supplemental Infection Control Strategies**

Parham Azimi^1*^, Zahra Keshavarz^1^, Jose Guillermo Cedeno Laurent^1^, Joseph G. Allen^1^

^1^ Harvard T. H. Chan School of Public Health, Department of Environmental Health

^*^ Corresponding Author; Email: [pazimi@hsph.harvard.edu](mailto:pazimi@hsph.harvard.edu)

# **APPENDIX A**

We developed a multi-zone transient Wells-Riley model using the following steps.

| $P_{infection}= 1-e^{-\bar{\mu}}$ | **Equation S. 1** |
| --- | --- |

$P_{infection}$: Probability of infection transmission in a school setup (-)

$\bar{\mu}$: Average number of quanta breathed by susceptible students during a typical school day (quanta)

We assumed:

1. Students stay continuously in each space during a period of exposure
2. The number of students in each space remains constant during an exposure period
3. The index case would not physically visit the recirculation spaces; rather, the generated quanta would reach to the recirculation space only through the HVAC system shared between the infector’s classroom and the recirculation spaces
4. Other transmission pathways of measles viruses such as direct contact or fomite are ignored

Therefore, $\bar{\mu}$ can be estimated from Equation S. ***2***.

| $\bar{\mu}=\frac{1}{N_{total}}\times\bar{p}\times\sum_{i} N_{i}\int_{0}^{\bar{t}_{i}} C_{qunata,i}\left( \tau\right)d\tau$ | **Equation S. 2** |
| --- | --- |

$N_{total}$: Total number of students in the schools during the infection period

$\bar{p}$: Average breathing rate of one student (m^3^ / hour)

$\bar{t}_{i}$: Average time that students spend in space *i* (hour)

$N_{i}$: Number of students in space *i* (-)

$C_{qunata,i}\left( \tau\right)$: Concentration of quanta in space *i*, $\tau$ hours after the index case enters the space (quanta / m^3^)

$C_{qunata,j}\left( \tau\right)$ for the infector’s classroom and the common area can be estimated by solving a well-mixed mass balance equation for each of these two spaces as demonstrated in Equation S. ***3*** - Equation **S. *7***.

| $\frac{dC_{quanta,j}(\tau)}{d\tau}=\lambda C_{quanta,out}(\tau)-K_{total,j}C_{quanta,j}(\tau)+\frac{Iq}{V_{j}}$ | **Equation S. 3** |
| --- | --- |

$I$: Number of index cases

$q$: Quanta generation rate (quanta / hour)

$V_{j}$: Volume of space $j$ – either infector’s classroom or common area – (m^3^)

$K_{total,j}$: Total removal rate of measles viruses in space $j$ – either infector’s classroom or common area – (per hour)

$\lambda$: Air exchange rate in in space $j$ – either infector’s classroom or common area – (per hour)

$C_{quanta,out}(\tau)$: Concentration of quanta in outdoor air (quanta / m^3^)

We assumed the concentration of quanta in outdoor air is zero; therefore, Equation S. ***3*** for the infector’s classroom and common area can be solved as shown in the following steps.

| $\frac{dC_{quanta,j}(\tau)}{C_{quanta,j}(\tau)-\frac{Iq}{V_{j}K_{total,j}}}=-K_{total,j}.d\tau$ | **Equation S. 4** |
| --- | --- |
| $\int_{C_{quanta,j}(\tau=0)}^{C_{quanta,j}(\tau)} \left( \frac{dC_{quanta,j}(\tau)}{C_{quanta,j}(\tau)-\frac{Iq}{V_{j}K_{total,j}}} \right)=\int_{\tau=0}^{\tau} \left( -K_{total,j}.d\tau\right)$ | **Equation S. 5** |
| $\ln\left( C_{quanta,j}(\tau)-\frac{Iq}{V_{j}K_{total,j}} \right)-\ln\left( -\frac{Iq}{V_{j}K_{total,j}} \right)= -K_{total,j}\tau$ | **Equation S. 6** |
| $C_{qunata,j}\left( \tau\right)=\frac{Iq}{V_{j}K_{total,j}}\left( 1-e^{-K_{total,j}\tau} \right)$ | **Equation S. 7** |

The format of the mass balance equation for the recirculation area would be different as we assumed the infectious bio-aerosols could reach to this area only through the HVAC air recirculation from the infector’s classroom as demonstrated in Equation S. ***8***

| $\frac{dC_{quanta,recir}(\tau)}{d\tau}=-K_{total,recir}C_{quanta,recir}(\tau)+\frac{F_{class-recir}C_{quanta,class}\left( \tau\right)}{V_{recir}}$ | **Equation S. 8** |
| --- | --- |

$C_{quanta,recir}\left( \tau\right)$: Concentration of quanta in the recirculation area

$K_{total,recir}$: Total removal rate of measles viruses in recirculation space (per hour)

$C_{quanta,class}\left( \tau_{n} \right)$: Concentration of quanta in infector’s classroom (quanta / m^3^)

$F_{class-recir}$: Transport coefficient of quanta from the infector’s classroom to the recirculation area (m^3^ / hour)

$V_{recir}$: Volume of recirculation area (m^3^)

Equation S. ***8*** shows the changes in quanta concentration the recirculation area is a function of concentration of quanta in the infector’s classroom; therefore, we adopted a discrete time-varying mass balance approach to solve Equation S. ***8*** similar to Azimi et al. (1) as demonstrated in Equation S. ***9***.

| $\frac{C_{quanta,recir}\left( \tau_{n} \right)-C_{quanta,recir}\left( \tau_{n-1} \right)}{\Delta\tau}=-K_{total,recir}C_{quanta,recir}\left( \tau_{n-1} \right)+\frac{F_{class-recir}C_{quanta,class}\left( \tau_{n-1} \right)}{V_{recir}}$ | **Equation S. 9** |
| --- | --- |

$\Delta\tau$: Time step interval, which is considered one minute in this model (hour)

The concentration of quanta in the infector’s classroom and recirculation area at the beginning of the simulation (i.e. $C_{quanta,recir}\left( \tau_{0} \right)$ and $C_{quanta,class}\left( \tau_{0} \right)$) assumed to be zero, the concentration of quanta in the infector’s classroom at any time step was estimated using Equation S. ***7***, and $F_{class-recir}$ was assumed to be constant during the school day and calculated from Equation S. ***10***.

| $F_{class-recir}=Q_{return,class}f_{recir}f_{runtime}\left( 1-\eta_{filter} \right)\times\frac{Q_{supply,recir}}{Q_{supply,total}}$ | **Equation S. 10** |
| --- | --- |

$Q_{return,class}$: Return airflow rate of the infector’s classroom (m^3^/hour)

$f_{recir}$: Fraction of recirculated air volume to total airflow capacity of HVAC system

$f_{runtime}$: Runtime fraction of HVAC system

$\eta_{filter}$: Removal efficiency of HVAC air filter

$Q_{supply,recir}$: Supply airflow rate of the recirculation space (m^3^/hour)

$Q_{supply,total}$: Total supply capacity of HVAC system (m^3^/hour)

The total removal rate of measles viruses in each space ($K_{total,i})$ was estimated by summing the removal rates of five infection elimination mechanisms as shown in Equation S. ***11***.

| $K_{total,i}=\lambda_{infilteration,i}+K_{deposition,i}+K_{ventilation,i}+K_{filtration,i}+K_{purification,i}$ | **Equation S. 11** |
| --- | --- |

$\lambda_{infilteration,i}$: Natural air ventilation rate or infiltration air exchange rate in space i (per hour)

$K_{deposition,i}$: Deposition rate of measles particles in space *i* (per hour)

$K_{ventilation,i}$: Mechanical ventilation rate of HVAC system in space *i* (per hour)

$K_{filtration,i}$: Infectious particle removal rate due to filtration in space *i* (per hour)

$K_{purification,i}$: Removal rate of infectious particles by standalone air handling units (AHU) or air purifiers in space *i* (per hour)

The removal rates droplet nuclei containing measles viruses due filtration ($K_{filtration,i}$), ventilation ($K_{ventilation,i}$) and air purification ($K_{purification,i}$) for space *i* – infector’s classroom, recirculation area, and common area – were estimated from Equation S. ***12*** - Equation **S. *14***.

| $K_{filtration,i}= \frac{f_{runtime}\times f_{recir}\times\eta_{filter}\times Q_{return,i}}{V_{i}}$ | **Equation S. 12** |
| --- | --- |
| $K_{ventilation,i}= \frac{f_{runtime}\times(1-f_{recir})\times Q_{return,i}}{V_{i}}$ | **Equation S. 13** |
| $K_{purification,i}= \frac{f_{runtime, AP,i}\times CADR_{AP,i}}{V_{i}}$ | **Equation S. 14** |

$Q_{return,i}$: Return air flow rate of HVAC system in space i (m^3^/hour)

$V_{i}$: Volume of space *i* (m^3^)

$f_{runtime, AP}$: Runtime fraction of air purifier in space *i*

$CADR_{AP,i}$: Clean air delivery rate of air purifier in space *i* (m^3^/hour)

# **APPENDIX B**

To back-calculate the quanta generation rate for primary schools, we relied on Riley et al’s study, in which the authors describe a measles outbreak in an elementary school in upstate New York, U.S. that occurred in the spring of 1974 (2). A total of 868 students attended the elementary school during the outbreak, out of which 28 of them were infected in the first generation of the outbreak. For secondary schools, we relied on a study by Chen et al., in which the authors described the characteristics of an outbreak in a high school in Illinois, U.S., in 1985 (3). A total of1873 students were enrolled at the high school during the outbreak, out of which, 69 cases were infected during the first and only generation of the outbreak. In both cases, measles was introduced to the school by one index case who did not use the school bus to commute to school. In the elementary school case study, the index case was a second-grade female student who became sick on Thursday, April 25^th^, 1974, and who attended all of her classes before any of her symptoms appeared. In the high school case study, the index case was a 16-year-old female student who had mild symptoms on Friday, April 12^th^, 1985 and attended all of her classes on that Friday and on the next Monday, April 15^th^, 1985 although a rash had appeared.

Both of the school records had shown high measles vaccination coverage before the outbreaks. In the elementary school, 97% of the students were vaccinated (VR = 0.97). We assumed all of the vaccinated students in the elementary school only had received one dose of measles vaccine because (i) the study did not report the number of students with two doses of measles vaccine and (ii) the outbreak happened in 1974 when receiving 2-dose vaccinations was not popular (as a piece of evidence, none of the studies before 1980 summarized in Tables 5 and 6 in the main manuscript, reported records of 2-dose measles vaccinations). In the high school case study, 99.7% of students were vaccinated before the outbreak, out of which approximately 70% and 30% of students had received one dose and two doses of the measles vaccine, respectively.

As demonstrated in Appendix A, in the developed model, we considered three spaces or microenvironments within a school building including the infector’s classroom, the recirculation area, and the common area. Therefore, we considered the same three microenvironments for the two studied schools during the back-calculation process. The average number of students in the elementary and high school classrooms were 24 and 30, respectively. In the elementary school case study, the ‘Main’ area, containing the classrooms for the first-, second-, and most of third-grade (except two classrooms) students, had its own air handling system. The infector’s classroom was located in the ‘Main’ area in the elementary school study. In the high school case study, all traditional classrooms were located in an area of the school named ‘Wing A’ using one air handling system that only served the classrooms. Moreover, in both case studies, students spent their lunch break in the schools’ cafeterias during the outbreaks, while no other common event or gathering was reported on those days. In the elementary school case study, all first-, second-, and third-graders had lunch together, while in the high school case study, there were three lunchtimes. Therefore, for the high school outbreak, we assumed one-third of students had lunch together during each lunch period.

Table S. ***1*** demonstrates our primary estimates and ranges for the risk model parameters. Most of the model variables were reported directly in the studies by Riley et al. and Chen et al. ; however, some of the parameters were not reported or measured during the outbreaks. In these cases, we considered a range for the model variables as shown in Table S. ***1***. We also chose a ‘best estimate’ or ‘primary estimate’ for each model parameter reflecting our finest estimations of that variable for use in the sensitivity analysis (Appendix D) as well as for demonstration purposes.

**Table S. 1.** Summary of outbreak characteristics in primary and secondary representative schools used in quanta generation rate (q) back-calculation process (Table 2 main manuscript)

| **Parameter** | **Primary School**  **Best-Estimate [Range]** | **Secondary School**  **Best-Estimate [Range]** | **Reference** |
| --- | --- | --- | --- |
| No. of enrolled students during outbreaks | 868 | 1873 | Literature^[1]^ |
| No. of first generation infected cases | 28 | 69 | Literature^[1]^ |
| No. of index case/s | 1 | 1 | Literature^[1]^ |
| Infection period in school (day) | 3 | 4 | Literature^[1]^ |
| Portion of unvaccinated students | 3.3% | 0.3% | Literature^[1]^ |
| Portion of students with 1-dose vaccination | 96.7% | 70.9% | Literature^[1]^ |
| Portion of students with 2-dose vaccination | 0.0% | 28.8% | Literature^[1]^ |
| No. of students in infector’s classroom | 24 | 30 | Literature^[1]^ |
| No. of students in recirculation area | 592 | 1843 | Literature^[1]^ |
| No. of students in common area | 664 | 1873 | Literature^[1]^ |
| Average time spent in classroom/s (mins) | 280 [270-290] ^[2]^ | 340 | Literature^[1]^ |
| Average time spent in recirculation area (mins) | 280 [270-290] ^[2]^ | 340 | Literature^[1]^ |
| Average time spent in common area (mins) | 20 [10-30] ^[2]^ | 70 | Literature^[1]^ |
| HVAC system runtime fraction | 1 | 0.768 | Literature^[1]^ |
| Recirculated air fraction | 0.438 | 0.05 | Literature^[1]^ |
| Supply airflow rate of one classroom (m^3^/min) | 28.3 | 8.5 | Literature^[1]^ |
| Total HVAC system capacity (m^3^/min) | 1019.4 | 595 | Literature^[1]^ |
| Air filter removal efficiency (%) | 12 | 12^[3]^ [10.5-42.2] | Literature^[4]^ |
| Occupancy density of classroom (m^2^/person) | 4 [3-5] | 4 [3-5] | DOE^[5]^ |
| Volume of recirculation area (m^3^) | 13832 [10374-17290] | 33600 [25200-42000] | Estimated^[6]^ |
| Occupancy density of common area (m^2^/person) | 1.39 [1.04-1.74] | 1.39 [1.04-1.74] | DOE^[5]^ |
| Inhalation rate (m^3^/day) | 12.96[11.34-14.53] | 15.53 [13.93-17.45] | EPA^[7]^ |
| Deposition rate of measles bio-aerosols (1/hour) | 1.7 [1.0-2.7] | 1.7 [1.0-2.7] | Literature^[4]^ |
| Natural ventilation rate (1/hour) | 0.31 [0.12-0.49] | 0.31 [0.12-0.49] | DOE^[8]^ |

1. **Cells highlighted in light gray are the model parameter values based on the information reported in Riley et al. (1978) and Chen et al. (1989) case studies** (2,3)**, while the dark gray colored cells contain variable values based on other existing studies**
2. **Assuming a similar lunchtime as the Chen et al. (1989) case study ±50%**
3. **For the primary estimate we considered the reported removal efficiency in Riley et al. (1978)** (2)
4. **Azimi and Stephens (2013), Table 4; assuming MERV4 and MERV 7 for the air filters** (4)
5. **U.S. Department of Energy commercial reference building models of the national building stock report,** (5)**; the average density of students in educational buildings (±25%)**
6. **For the elementary school estimated based on the HVAC total capacity versus supply air flow of each classroom and for the high school calculated based on the of occupancy density of classrooms and the school’s floor plan**
7. **U.S. EPA,** (6)**; Interpolated from the reported inhalation rates of children in various age ranges in the Exposure Factors Handbook: 2011 Edition, Table 6.23**
8. **U.S. Department of Energy commercial reference building models of the national building stock,** (5)**; Table A-2, primary and secondary education buildings**

Students at the elementary school spent 300 minutes in regular classes and lunchtime period and students at the high school spent 410 minutes in 10 five-minutes exchange periods, 3 twenty-minutes lunchtimes, and six 50-minutes regular classes. In both case studies, we assumed that the index cases stayed in contact with their classmates most of the time during regular class periods and interacted with all students in the school cafeterias and hallways during the lunchtime and class breaks. Riley et al. did not report the lunchtime and class break durations in the elementary school; therefore, we assumed a 20 minutes lunch break (similary to the study of Chen et al.) as our primary estimate for the time spent in the common area changing between 10 and 30 minutes. For the high school case study, we chose the combination of all class breaks and the lunchtime (i.e., 70 minutes) as our best estimate of the time that the index case spent in the common area.

The heating, ventilation, and air conditioning (HVAC) systems of both schools were recirculating the indoor air and passing it through air filters. They also provide fresh outdoor air to the classrooms at various rates depending on the outdoor air temperature. In the elementary school, the HVAC system ran all the time and supplied 28.3 m^3^/min of mixed recirculated and fresh air to each classroom with a total capacity of 1019.4 m^3^/min. The portion of provided outdoor air in the elementary school was 100%, 40%, and 28.6% (average of 56.2%) on one, two, and three days before the disease symptoms appeared, respectively. In the high school, the HVAC system provided 8.5 m^3^/min of mixed recirculated and fresh air to each classroom in Wing A, but hallways have neither ventilation outlets nor windows. The total HVAC system capacity of the high school was not reported; therefore, we estimated the total HVAC capacity of 595 m^3^/min for the ‘Wing A’ heating and cooling system as this part of the school had 70 rooms (shown on the school floor plan) and all of them were served by one system. The high school HVAC system ran during the first 45 minutes of each period and then was turned off during the last 5 minutes of the class-time periods and during the five-minute exchange periods. We assumed a similar trend for HVAC system runtime during the lunchtime periods (i.e., running during the first 15 minutes and then not runningin the next 10 minutes) and calculated the total HVAC system runtime of 76.8% (i.e., 315 mins / 410 mins) in the high school. Moreover, in the high school, the vents supplied 90-100% fresh outdoor air on three days before the outbreak started.

As shown in Equation S. ***12*** and Equation **S. *13*** in Appendix A, the bio-aerosol removal efficiencies of the air filters ($\eta_{filter}$) and the volume of each microenvironment ($V_{i}$) are required for estimating the filtration and ventilation rate of the schools’ HVAC systems. Riley et al. reported the air filter efficiency of 12% for the HVAC system in the ‘Main’ area at the elementary school. We assumed a similar air filter efficiency (i.e. 12%) as the best estimate for the high school HVAC system and considered a range between 10.5% and 42.2% assuming MERV4 and MERV7 were deployed in the high school HVAC system, respectively, based on the study by Azimi and Stephens

(4).

Neither of the case studies reported the volume of the classrooms, the recirculation area, or the common area, therefore, we adopted a variety of approaches to estimate the volume of various spaces in the schools. For the classrooms and common areas in both schools, we assumed an average floor area per person of 4 m^2^/person (± 25% as the range) and 1.39 m^2^/person (± 25% as the range), respectively, based on the commercial reference building models of the national building stock report published by the U.S. Department of Energy (DOE) (5). We assumed the floor area per person of a typical cafeteria in an educational building as the representative of the common area occupancy density because in both cases the lunch breaks were the primary common event among all students of the studies schools. The volume of the recirculation area for the elementary school was estimated based on the ratio of provided air to one classroom and the total capacity of the elementary HVAC system (i.e., 28.3 m^3^/min / 1019.4 m^3^/min) and for the high school was estimated simply by multiplying the number of rooms in ‘Wing A’ (i.e., 70) to the volume of one classroom.

The average breathing rate of one student in the elementary and high schools was estimated based on the data provided in the U.S. EPA Exposure Factors Handbook: 2011 Edition, Table 6.23 (6). The handbook did not report the inhalation rates for the age groups that we used in this study (i.e., between 6 -14 years old for primary and 14 -18 years old for secondary students); therefore, we used an interpolation approach to estimate the inhalation rate of the students in the studied ages. The average ages of students in the elementary and high schools were 10 and 16 years old, respectively. The EPA handbook reported the average (range) inhalation rates for children between 6 -11, 11-16, and 16 -21 are 11.96 (9.98-13.42), 15.17 (14.29-16.98), and 16.25 (14.29-18.29), respectively. We deployed a linear regression line between the data points to estimate the average inhalation rate of primary (10 years old) and secondary (16 years old) students as demonstrated in Figure S. ***1***. It is noticeable that Riley et al. used an inhalation rate of 8.15 m^3^/day for students in the elementary school, which was significantly lower than our average assumption of 12.96 m^3^/day used in this study. However, because Riley et al. did not actually measure this parameter during the outbreak, we used the recent inhalation rate estimations for students.


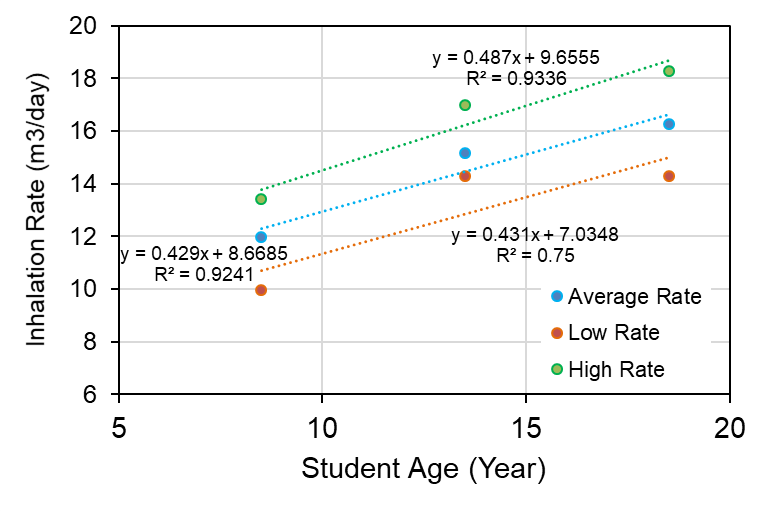


**Figure S. 1**. Estimating the inhalation rate based on EPA handbook 2011 edition, Table 6.23

The deposition rate of infectious droplet nuclei and natural ventilation rates in various microenvironments in the schools were not measured or reported originally during the outbreaks. The deposition rate of droplet nuclei containing measles viruses was assumed to be similar to the deposition rate of influenza bio-aerosols reported in Azimi and Stephens’s study changing between 1 and 2.7 per hour with the average value of 1.7 per hour (4). For the natural ventilation rate, we again relied on the commercial reference building models of the national building stock report (5). In the DOE report, the infiltration rates of various microenvironments were varied between 0.12 and 0.49 air exchanges per hour (ACH) for modeled primary and secondary education buildings with central forced air systems. We selected the infiltration rate of a ‘MULT_CLASS’ on the first floor of a typical elementary school as our best estimate.

It is noticeable that because we back-calculated the quanta generation rate from actual epidemiology studies, any simplification that we deployed during the model development and variable estimation will be considered automatically in the quanta generation estimates. We also determined the boundaries of the quanta generation rates for the primary and secondary schools based on the low and high values of the model parameters.

# **APPENDIX C**

We developed a nationwide school model that represents the building and epidemiological characteristics of a majority of elementary and high schools in the U.S. The combination of the nationwide representative School Building Archetype (SBA) model and the developed multi-zone transient Wells-Riley model was used to estimate the range of measles risk that students are facing in U.S. schools. The SBA model considers two sets of parameters for primary and secondary schools to examine the impacts of students’ age and activity patterns on the risk model results. The definition of primary and secondary education schools was based on the structure of education in the United States shown in Figure S. ***2*** (7). We considered students less than 14 years old in elementary (primary) and students between 14 and 18 years old in high (secondary) schools.


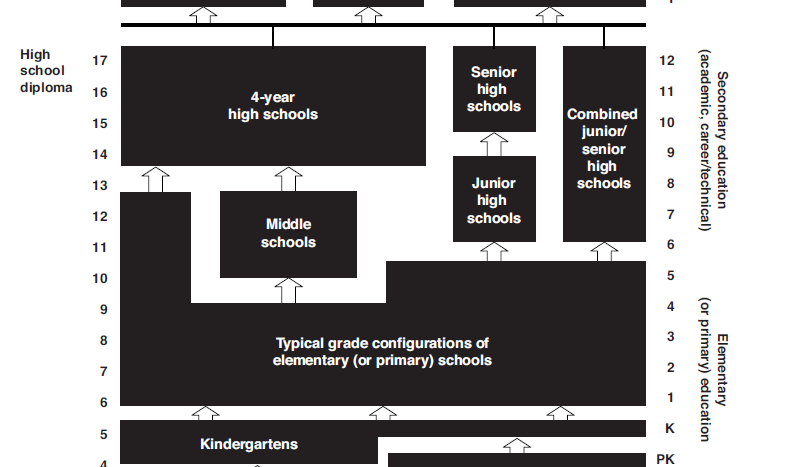


**Figure S. 2.** Structure of education in US for students between 6 and 18 years old (7)

We assumed a teacher self-contained format for the primary schools and a departmentalized format for the secondary schools, as the majority of elementary schools in the United States have self-contained classrooms (8,9). Therefore, the estimates of quanta generation rate back-calculated from the teacher self-contained elementary and the departmentalized high school case studies in Appendix B were used for primary and secondary schools in the developed SBA model, respectively. Other model variables were either assumed or culled from existing resources.

The latest number of educational institutions in the U.S. reported by the Department of Education, National Center for Education Statistics in 2019 shows 88,665 primary schools and 26,986 secondary schools were registered nationwide in the 2015-16 school year. Moreover, the survey reported 16,511 combined and 691 other educational institutions in the U.S., which we did not include in the SBA model (7). The number of enrolled students in a U.S. school is another variable changing significantly from 1 to more than 8,500 students (10). In this study, our best estimate of the number of students in a regular public school was 513 and 854 students for a primary and secondary school, respectively, based on the National Center for Education Statistics report in 2012 (10). We also considered the 5^th^ and 95^th^ percentiles of student membership size of a regular public school in different states as the range of student enrollment in U.S. schools (10).

Next, we culled the epidemiological characteristics of measles viruses from existing studies. We assumed one index case starts the outbreak, which has been the case in almost all of the reported measles outbreaks in U.S. schools. We also assumed a *school* *infectious period* (defined as the period during which the index case spreads the measles pathogens among susceptible students) of 3 days as our primary estimate of the variable (ranging between 2 and 4 days). The primary estimate and the range for the school infectious period were selected based on several studies suggesting the infectious period of measles starts several days before the symptoms appear (11,12) and the infectivity is greatest during 3 days before the onset of a rash (13). This assumption was also based on an expectation that the index case would avoid attending school after the symptoms appear.

To estimate the measles vaccine coverage among primary and secondary students in the U.S., we relied on a combination of reports. The measles vaccine coverage in the U.S. has stayed approximately constant since the Vaccines for Children (VFC) program began in 1994 (14). Centers for Disease Control and Prevention (CDC) has annually reported the national and regional ≥ 1-dose vaccination coverage among children aged 19–35 months as well as ≥ 2-dose vaccination coverage among adolescents aged 13–17 years. The vaccination coverage among children aged 19-35 months in 2017, who havereceived 1 dose or more of the measles vaccine, was 91.5% (CI: 90.6% - 92.3%) which has not changed significantly since 1996 (15,16). The average estimated nationwide vaccination coverage among adolescents aged 13–17 years who have received two doses or more of measles vaccine, was 92.1% (91.3% – 92.8%) in 2017 ranged between 89.6% and 92.1% between 2013 and 2017 (17–19). Lopez et al. demonstrated the ≥ 2-dose vaccination coverage of 7-year old children in the U.S. was ~90% between 2006 and 2012 (20). Moreover, based on the CDC’s recommended immunization schedule, children and adolescents aged 18 years or younger in the United States should receive the 1^st^ dose of measles, mumps, rubella (MMR) vaccine when they are between 12- and 15 months and the second dose when they are between 4 and 6 years old. These reports demonstrated that the portion of students in the U.S. who have received *only* one dose of measles vaccine is nominal. Therefore, for the SBA model, we assumed 91% (ranged between 90% - 92%) of 6 - 18 year old students in both primary and secondary education institutions in the U.S. have received ≥ 2-dose measles vaccine and the rest of the students are unvaccinated. Moreover, similar to assumptions in Appendix B, we assumed that approximately 1% of ≤14 year old students and 5% of 14-18 year old students who have received more than two doses of the measles vaccine remain susceptible to measles (21–26).

For the number of students in a typical primary or secondary education classroom, we relied on the latest Schools and Staffing Survey (SASS) reported by the National Center for Education Statistics (NCES) in which the average class sizes of public primary, middle, and high schools are listed by classroom type and state in the 2011–12 school year (27). The report listed two types of classrooms including departmentalized instruction and teachers in self-contained classes. For the primary and secondary education classrooms, we considered the average sizes of teacher-self-contained and departmentalized classes equal to 21 students (5^th^ and 95^th^ percentiles of 18 and 26 students) and 23 students (5^th^ and 95^th^ percentiles of 18 and 30 students), respectively.

In the SBA model, we assumed if a school uses a central-forced-air system for cooling or heating, only one system serves all classrooms; therefore, the number of students in the recirculation area was estimated equal to the total number of enrolled students minus the number of students in the infector’s classroom. In the lack of a reliable nationwide survey on how HVAC systems serve indoor spaces in U.S. schools, this assumption was taken for simplicity. Moreover, we assumed all students in the SBA model are exposed to droplet nuclei emitted from the index case in the common area. We believe it is a reasonable assumption because several studies have shown the measles viruses stay viable and infectious for up to two hours in an indoor environment even after the index case leaves the space (28–30) and there is a high chance that all students appear in the common areas such as a cafeteria within two hours after an infector’s presence, particularly during lunchtime.

The volume of classrooms and recirculation and common areas were estimated based on the number of students and the occupancy density of the spaces. Similar to the back-calculation process, we assumed the occupancy density of the classrooms and recirculation area to be 4 m^2^/person (ranged between 3 and 5) and the common area to be 1.39 m^2^/person (ranged between 1.04 and 1.74) (5). It is noticeable that we considered cafeteria as the representative of the common area in this SBA model because:

1. The time that students usually spend in the hallways are insignificant in comparison to other school microenvironments (2),
2. Hallway-contact models demonstrated a limited number of students would have a chance to directly interact with the index case (3)
3. Having one meal in a school’s cafeteria is a common daily student-gathering event in most U.S. schools.

In the SBA model, a microenvironment volume was estimated using Equation S. ***15***.

| $V_{k}=D_{k}\times H_{k}\times N_{k}$ | **Equation S. 15** |
| --- | --- |

$V_{k}$: Volume of space k, which is either a classroom, recirculation, or common area

$D_{k}$: Occupancy density of space k

$H_{k}$: Height of space k, which assumed to be 4 meters for all spaces in the SBA model

$N_{k}$: Number of students in space k

Students in the U.S. on average spend 6.64 hours in school daily with 5^th^ and 95^th^ percentiles of 6.25 and 7.05 hours, respectively (31). For the SBA model, we considered a typical school day that students spend most of their time in regular classes. In this model, similar to our assumptions during the back-calculation process, we assumed students stay in classrooms all the time except during the lunch break. We assumed, on average, a lunch break would take 20 minutes (ranged between 15 and 30 minutes) and 30 minutes (ranged between 20 and 45 minutes) in a primary and secondary school, respectively (32). We considered a school academic year of 290 days for the representative model, which starts in mid-August and ends in late May creating a 200-day (from October to mid-April) heating season and a 90-day cooling season.

Schools in the U.S. use different types of heating and cooling systems. Winiarski et al. analyzed the content of the 2003 Commercial Building Energy Consumption Survey (CBECS) to determine the most common HVAC systems used in 15 different building types, including primary and secondary schools (33,34). They divided the regular HVAC systems into two categories of post-1980 and pre-1980 buildings. Later in 2018, they updated the list based on the most recent CBECS in 2012 reporting the typical HVAC systems in U.S. buildings constructed after 1990 (35,36). We divided the summarized systems into three main categories of “*Central-Forced-Air*” and “*Ductless-with-Air-Filter*” and “*Ductless-without-Air-Filter*”. The systems categorized as *Central-Forced-Air* would recirculate the indoor air between the infector’s classrooms and the recirculation area in the modeled schools, the *Ductless-with-Air-Filter* systems would recirculate the air only within each room, and *Ductless-without-Air-Filter* systems use heated or cooled liquids (e.g. oil or water) instead of air to control the temperature of the indoor spaces. We also assumed *Central-Forced-Air* and *Ductless-with-Air-Filter* systems could filter the infectious droplet nuclei in the infector’s classroom or other indoor spaces. Table S. ***2*** demonstrates how the different HVAC system types are divided into the mentioned categories based on their ability for recirculating and filtrating the indoor air. For example, summarized data in Table S. ***2*** shows 50%, 25%, and 25% of schools utilizing ‘District Heating and Cooling Systems’ were considered as Central-Forced-Air, Ductless-with-Air-Filter, and Ductless-without-Air-Filter HVAC systems, respectively.

**Table S. 2.** Categorizing HVAC system types based on the ability to recirculate and filter the indoor air

| **HVAC System Type** | **Central-Forced-Air** | **Ductless-w/-Air-filter** | **Ductless-w/o-Air-filter** |
| --- | --- | --- | --- |
| District Heating and Cooling Systems | 50% | 25% | 25% |
| Systems with Heat Pump (HP) | 50% | 25% | 25% |
| Systems with Boiler | 50% | 25% | 25% |
| Systems with Furnace | 100% | 0% | 0% |
| Systems with Chiller | 50% | 25% | 25% |
| Packaged Central Units (PCU) | 0% | 100% | 0% |
| Packaged Air Conditioning Units (PACU) | 0% | 100% | 0% |
| Packaged Heating Units (PHU) | 0% | 100% | 0% |
| Residential-Type Central Air Conditioner (RES CAC) | 100% | 0% | 0% |
| Residential Air Conditioner (RES. AC) | 100% | 0% | 0% |
| Individual Space Heater (ISH) | 0% | 50% | 50% |
| Individual Room Air Conditioner (IRAC) | 0% | 100% | 0% |
| Other systems | 50% | 25% | 25% |
| No HVAC system | 0% | 0% | 100% |

Table S. ***3*** summarizes the proportion of Central-Forced-Air, Ductless-with-Air-Filter, and Ductless-without-Air-Filter heating and cooling systems used in U.S. school buildings post- 1980, between 1980 and 2003 and between 1990 and 2012. Table S. ***3*** also demonstrates our best estimates of the proportions of heating and cooling system types deployed in U.S. schools based on the average age of the school buildings. NCES in 1999 reported 28% of schools in the U.S. were built before 1950, 62% between 1950 and 1985, and 10% after 1985 with an average building age of 42 years (37). The latest report of Condition of America’s Public School Facilities released by NCES in 2014 noted the average age of school buildings in the U.S. is 44 years, but they did not report the school buildings’ year of built (38). In the lack of other reliable sources for the building age of schools and considering the fact that the average school building age did not increase significantly between 1999 and 2014, we assumed the school buildings that were built before 1950 should have been replaced with new ones. Therefore, we assumed approximately 30% of the schools are built after 1990, 10% around 1985 (i.e. between 1980 and 90), and 60% before 1980.

**Table S. 3.** Summary of HVAC system types in US schools based on 2003 and 2012 Commercial Building Energy Consumption Survey

|  | | **Pre-1980 schools based on** (34) | | **1980 - 1990 schools based on** (34) | | **Post-1990 schools based on** (36) | | **Best estimate for all schools** | |
| --- | --- | --- | --- | --- | --- | --- | --- | --- | --- |
|  |  | **Heating** | **Cooling** | **Heating** | **Cooling** | **Heating** | **Cooling** | **Heating** | **Cooling** |
| Primary School | Central-Forced-Air | 50% | 21% | 45% | 43% | 23% | 33% | 41% | 26% |
|  | Ductless-w/-Air-filter | 38% | 69% | 42% | 47% | 67% | 58% | 47% | 63% |
|  | Ductless-w/o-Air-filter | 13% | 10% | 14% | 11% | 11% | 10% | 12% | 10% |
| Secondary School | Central-Forced-Air | 52% | 31% | 55% | 40% | 21% | 44% | 43% | 35% |
|  | Ductless-w/-Air-filter | 30% | 60% | 31% | 41% | 67% | 45% | 41% | 54% |
|  | Ductless-w/o-Air-filter | 18% | 9% | 14% | 20% | 12% | 11% | 16% | 11% |

Unfortunately, we are not aware of a reliable source for the recirculation rate of HVAC systems in school classrooms. In one of the few studies in this area, Polidori et al. measured the average recirculation rate of 6.4 (ranged between 3.3 and 8.5) per hour in nine California classrooms (39). This study was used later in a study by Chan et al. (40) to estimate the effect of ventilation and filtration on chronic health risks in U.S. schools as the representative air recirculation rate in a typical classroom. Moreover, we assumed the HVAC system runtime of 100% for heating and cooling systems capable of recirculating and filtrating the indoor air, as well as for ventilation systems providing fresh air to the modeled schools’ spaces.

To estimate the outdoor air ventilation rate in the school buildings, we relied on the ASHRAE Standard 62.1-2016 (41). The standard suggests a variety of *default* ventilation rates for different types of rooms (not including cafeterias or hallways) in educational facilities ranging between 4.0 and 9.5 L/s-person, which were considered as the range of outdoor ventilation rate of the infector’s classroom and the recirculation area in the SBA model. Our best estimate of ventilation rate in the infector’s classroom and the recirculation area was assumed to be 6.7 L/s-person, which was equal to the ASHRAE default ventilation rate for a classroom with students above 9 years of age. Moreover, as student gatherings in the cafeteria or dining room of schools were considered as primary events, where the susceptible students are exposed to infectious aerosols, we used the ASHRAE standard recommended ventilation rates for the cafeteria and dining rooms as the ventilation rates of common areas in the SBA model. The ASHRAE Standard 62.1-2016 suggests default ventilation rate of 4.7 and 5.1 L/s-person for cafeteria and restaurant dining rooms, respectively, which was assumed the range of ventilation rate in the common area and our best estimate was simply the average of these two values. The ASHRAE standard did not recommend any ventilation rate for the corridors as one of the occupancy categories.

EPA’s “Tool for School” program requires all schools to at least have air filters with Minimum Efficiency Reporting Value (MERV) of 8 in all HVAC application, while the National Air Filtration Association (NAFA) recommends air filters between MERV 8 and 13 for schools (42,43). In this study, we considered MERV11 filters as our primary scenario and considered a range of air filters between MERV8 and MERV13 for the SBA model. The bio-aerosols’ removal efficiencies of various air filters were adopted from the study by Azimi and Stephens assuming the values are similar for measles and influenza viruses (4).

Finally, natural ventilation, deposition, and inhalation rates in the SBA model were assumed similar to our assumptions in the quanta generation rate back-calculation process. The natural ventilation rates (infiltration rate) of the primary and secondary schools were assumed to be 0.31 per hour ranging between 0.12 and 0.49 per hour, based on the school representative model suggested by the U.S. Department of Energy (5). The deposition rate of droplet nuclei containing measles viruses in indoor spaces was assumed to be 1.7 per hour ranging between 1 and 2.7 per hour (4). The inhalation rate for primary (between 6 and 14 years old) and secondary (between 14 and 18 years old) students were assumed to be 12.96 m^3^/day (ranging between 11.34 and 14.53 m^3^/day) and 15.53 m^3^/day (ranging between 13.93 and 17.45 m^3^/day), respectively.

Similar to the back-calculation process, we chose a best (primary) estimate for each SBA model variable for the sensitivity analysis and demonstration purposes. In addition to a primary estimate, we considered a range for most of the SBA model variables. We applied a Monte-Carlo simulation with 10,000 iterations to account for the impacts of changes in the model parameter values on the transmission risk results. Each iteration represents the risk of measles transmission in one U.S. school setup. For the Monte-Carlo simulation, we culled the model variables from two decks of primary and secondary inputs with the same proportion as the ratio of primary and secondary educational institutions in the U.S. (i.e., 76.6% of iterations were from primary school inputs and 23.3% were from secondary school inputs) (7). A similar approach was adopted for the ratio of heating and cooling system types in the SBA model. The number of times that we selected each heating and cooling system type (i.e., central-forced-air systems and ductless HVAC systems with and without air filters) in the Monte-Carlo simulation was based on our best estimates of the proportion of the heating and cooling system types used in the U.S. schools as summarized in Table S. ***3***.

Table S. ***4*** summarizes the best estimates and ranges of the SBA model variables.

**Table S. 4.** Summary of best estimates and ranges of variables used in the nationwide representative School Building Archetype (SBA) model (Table 3 main manuscript)

| **Parameter** | **Primary School**  **Best-Estimate [Range]** | **Secondary School**  **Best-Estimate [Range]** | **Reference** |
| --- | --- | --- | --- |
| No. of educational institutions in US 2015-2016 | 88,665 | 26,986 | NCES^[1]^ |
| No. of Index case/s | 1 | 1 | Assumption |
| Quanta generation rate (quanta / hour) | 1925 [1185 - 3345] | 2765 [1430 - 5140] | This Study^[2]^ |
| No. of enrolled students before outbreak | 513 [175 - 825] | 854 [245-1394] | NCES^[3]^ |
| Infection period in school (day) | 3 [2 - 4] | 3 [2-4] | Literature^[4]^ |
| Portion of unvaccinated students | 9% [8% - 10%] | 9% [8% - 10%] | CDC^[5]^ |
| Portion of students with ≥ 2-dose vaccination | 91% [90% - 92%] | 91% [90% - 92%] | CDC^[5]^ |
| No. of students in infector’s classroom | 21 [18 -26] | 23 [18-30] | SASS^[6]^ |
| Occupancy density of classroom (m^2^/person) | 4 [3-5] | 4 [3-5] | DOE^[7]^ |
| Occupancy density of common area (m^2^/person) | 1.39 [1.04-1.74] | 1.39 [1.04-1.74] | DOE^[7]^ |
| Average time spent in school (mins) | 400 [375-425] | 400 [375-425] | SASS^[8]^ |
| Average time spent in common area (mins) | 20 [15-30] | 30 [20-45] | NFSMI^[9]^ |
| Heating and cooling periods in US schools (day) | H: 200 & C: 90 | H: 200 & C: 90 | Assumption^[10]^ |
| HVAC system type | See Table 4  (main manuscript) | See Table 4  (main manuscript) | CBECS ^[11]^ |
| HVAC recirculation rate in classrooms (per hour) | 6.4 [3.3–8.5] | 6.4 [3.3–8.5] | Literature^[12]^ |
| Outdoor air ventilation in classrooms (L/s-person) | 6.7 [4.0 – 9.5] | 6.7 [4.0 – 9.5] | ASHRAE^[13]^ |
| Outdoor air ventilation in common area (L/s-person) | 4.9 [4.7 – 5.1] | 4.9 [4.7 – 5.1] | ASHRAE^[13]^ |
| HVAC runtime for applicable systems | 1 | 1 | Assumption |
| Air filter removal efficiency (%) | 72% [44% - 86%] | 72% [44% - 86%] | NAFA^[14]^ |
| Infiltration rate (1/hour) | 0.31 [0.12 – 0.49] | 0.31 [0.12 – 0.49] | DOE^[7]^ |
| Deposition rate of measles bio-aerosols (1/hour) | 1.7 [1.0 – 2.7] | 1.7 [1.0 – 2.7] | Literature^[15]^ |
| Inhalation rate (m^3^/day) | 12.96 [11.34- 14.53] | 15.53 [13.93- 17.45] | EPA^[16]^ |

1. **U.S. Department of Education, National Center for Education Statistics (NCES),** (7)**; Table 105.50 “Number of educational institutions, by level and control of institution: Selected years, 1980–81 through 2015–16”**
2. **The method explained in “Back-calculating quanta generation rate” Section and results are provided in “Estimates of quanta generation rate” Section**
3. **U.S. Department of Education, NCES,** (10)**; Table 5 “Average student membership size of regular public elementary and secondary schools with membership, by instructional level, membership size of largest and smallest school, and state or jurisdiction: School year 2009–10”**
4. **Based on existing epidemiological literature** (11–13)
5. **Centers for Disease Control and Prevention** (15,17)
6. **U.S. Department of Education, NCES , Schools and Staffing Survey (SASS)** (27)**; Table 7. “average class size in public primary, middle, and high schools is listed by classroom type and state in school year 2011–12”**
7. **U.S. Department of Energy commercial reference building models of the national building stock,** (5)**; Appendix A**
8. **U.S. Department of Education, NCES, SASS,** (31)**; “Average number of hours in the school day and average number of days in the school year for public schools, by state: 2007–08”**
9. **National Food Service Management Institute** (32)
10. **200 days of heating season from October to mid-April and 90 days of cooling seasons in one school academic year**
11. **U.S. Energy Information Administration, Commercial Buildings Energy Consumption Survey** (34,36)
12. **Based on Polidori et al. and Chan et al. studies** (39,40)
13. **ASHRAE Standard 62.1-2016 Ventilation for Acceptable Indoor Air Quality (2016)** (41)
14. **National Air Filtration Association** (42)
15. **Based on Azimi and Stephens study** (4)
16. **U.S. EPA Exposures Factors Handbook** (6)

# **APPENDIX D**

We explored the sensitivity of the results to the changes in the developed Wells-Riley and SBA model variables. We divided the variables into three categories of biological-epidemiological, human interaction, and HVAC-and-building-related variables as demonstrated in Table S. ***5*** and Table S**. *6***. Table S. ***5*** shows our best estimates and ranges for each model parameter, while the percentage changes in the variable values from the primary estimates are shown in parenthesis. We also calculated the average percentage changes in the variable values which were used later in the sensitivity diagrams in Figure S. ***3***.

**Table S. 5.** Sensitivity analysis; changes in the developed Wells-Riley and SBA model variables

| Model Variables | | Best Estimate | | Low Estimates  (Change from best estimate) | | | High Estimates  (Change from best estimate) | | |
| --- | --- | --- | --- | --- | --- | --- | --- | --- | --- |
|  |  | Pri. School | Sec. School | Pr. School | Sec. School | Ave. change | Pr. School | Sec. School | Ave. change |
| Human Interaction | Total No. students | 513 | 854 | 175  (-66%) | 245  (-71%) | -69% | 825  (61%) | 1394  (63%) | 62% |
|  | No. students in classrooms | 21 | 23 | 18  (-14%) | 18  (-22%) | -18% | 26  (24%) | 30  (30%) | 27% |
|  | Time spent at school (min) | 400 | 400 | 375  (-6%) | 375  (-6%) | -6% | 425  (6%) | 425  (6%) | 6% |
|  | Time spent in common area (min) | 20 | 30 | 15  (-25%) | 20  (-33%) | -29 | 30  (50%) | 45  (50%) | 50% |
|  | Classroom occ. density (m^2^/person) | 4 | 4 | -25%  3 | -25%  3 | -25% | 25%  5 | 25%  5 | 25% |
|  | Common area occ. density (m^2^/person) | 1.39 | 1.39 | 1.04  (-25%) | 1.04  (-25%) | -25% | 1.74  (25%) | 1.74  (25%) | 25% |
| Biological-Epidemiological | Quanta generation rate (quanta / hour) | 1925 | 2765 | 1185  (-38%) | 1430  (-48%) | -43% | 3345  (74%) | 5140  (86%) | 80% |
|  | Infection period (day) | 3 | 3 | 2  (-33%) | 2  (-33%) | -33% | 4  (33%) | 4  (33%) | 33% |
|  | Vaccination coverage | 0.09 | 0.09 | 0.08  (-11%) | 0.08  (-11%) | -11% | 0.1  (11%) | 0.1  (11%) | 11% |
|  | Inhalation rate (m^3^/day) | 12.96 | 15.53 | 11.34  (-13%) | 13.93  (-10%) | -11% | 14.3  (12%) | 17.45  (12%) | 12% |
|  | Bio-aerosol deposition rate (1/hr) | 1.7 | 1.7 | 1  (-41%) | 1  (-41%) | -41% | 2.7  (59%) | 2.7  (59%) | 59% |
| HVAC-Building | Classroom recircu. rate (1/hr) | 6.4 | 6.4 | 3.3  (-48%) | 3.3  (-48%) | -48% | 8.5  (33%) | 8.5  (33%) | 33% |
|  | Classroom venti. rate (1/hr) | 6.7 | 6.7 | 4  (-40%) | 4  (-40%) | -40% | 9.5  (42%) | 9.5  (42%) | 42% |
|  | Common area vent. rate (1/hr) | 4.9 | 4.9 | 4.7  (-4%) | 4.7  (-4%) | -4% | 5.1  (4%) | 5.1  (4%) | 4% |
|  | Air filter removal efficiency | 0.72 | 0.72 | 0.44  (-39%) | 0.44  (-39%) | -39% | 0.859  (19%) | 0.859  (19%) | 19% |
|  | Infiltration rate (1/hr) | 0.31 | 0.31 | 0.12  (-61%) | 0.12  (-61%) | -61% | 0.49  (58%) | 0.49  (58%) | 58% |

Table S. ***6*** shows the absolute transmission risk of measles in both primary and secondary schools associated with the low and high ranges of the models’ variables demonstrated in Table S. ***5*** as well as their percentage change from the best estimate of measles transmission risk among all students (i.e. 3.5% Figure 2 in the main manuscript).

**Table S. 6.** Sensitivity analysis results; absolute and percentage changes in transmission risks of measles associated with changes in the developed Wells-Riley and SBA model variables

|  | Model Parameter | Absolute transmission risk among all students associated with | | Percentage changes in transmission risks from the best infection risk estimate of 3.5% among all students associated with | |
| --- | --- | --- | --- | --- | --- |
|  |  | Low variable values | High variable values | Low variable values | High variable values |
| Human Interaction | Total No. students | 7.6% | 2.3% | 118.03% | -33.80% |
|  | No. students in classrooms | 3.5% | 3.4% | -1.15% | -1.52% |
|  | Time spent at school | 3.3% | 3.6% | -6.33% | 3.16% |
|  | Time spent in common area | 3.4% | 3.5% | -2.42% | 1.05% |
|  | Classroom occupancy density | 4.0% | 3.0% | 15.37% | -13.29% |
|  | Common area occupancy density | 3.5% | 3.4% | 0.70% | -3.08% |
| Biological-Epidemic | Quanta generation rate | 2.2% | 5.3% | -37.52% | 51.37% |
|  | Infection period | 2.5% | 4.3% | -29.63% | 23.97% |
|  | Vaccination coverage | 3.1% | 3.8% | -10.17% | 7.5% |
|  | Inhalation rate | 3.5% | 3.4% | -10.82% | 7.69% |
|  | Bio-aerosol deposition rate | 3.8% | 3.1% | 7.43% | -11.52% |
| HVAC-Building | Classroom recirculation rate | 4.2% | 3.1% | 19.21% | -10.89% |
|  | Classroom ventilation rate | 3.7% | 3.2% | 5.58% | -7.72% |
|  | Common area ventilation rate | 3.5% | 3.4% | -1.25% | -1.48% |
|  | Air filter removal efficiency | 4.3% | 3.1% | 23.19% | -10.61% |
|  | Infiltration rate | 3.1% | 3.8% | 1.02% | -3.41% |

Figure S. ***3*** presents the results of the sensitivity analysis. In each diagram, the x- and y-axis values show the changes in the model variables and the transmission risks associated with those changes, respectively. The centers of the diagrams represent the variables and transmission risks associated with our best estimates, which are located at the origin of the coordinate plane (i.e., 0% - 0%). Each branch shows the relative variation of a model parameter from its primary estimate (i.e., average changes in Table S. ***5***) and the associated relative changes in the transmission risk estimates (i.e., percentage changes in Table S. ***6***). The lengths of the branches were limited to the ranges of the models’ variables.


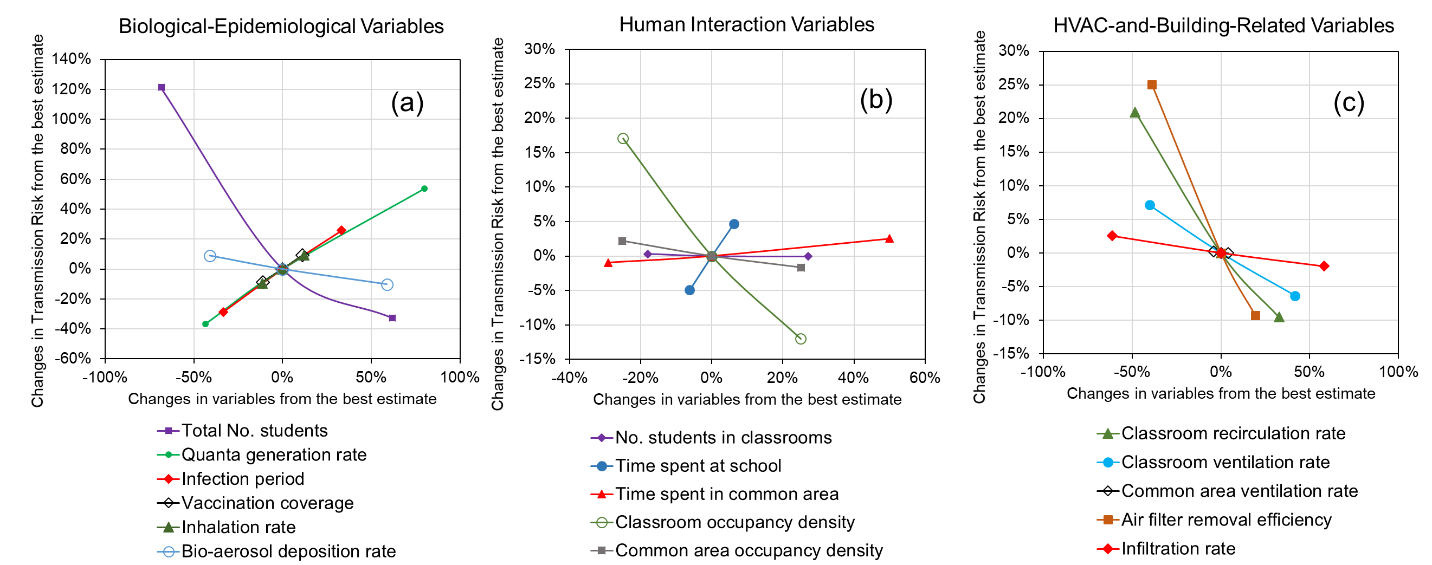


**Figure S. 3.** Sensitivity of the measles transmission model in U.S. schools to changes in (a) biological-epidemiological variables, (b) human-interaction-related parameters and (c) HVAC-building-related variables (Figure 4 main manuscript)

The sensitivity results show (i) the total number of enrolled students, quanta generation rate, and infection period among biological-epidemiological variables; (ii) occupancy density of the infector’s classrooms among human-interaction-related parameters; and (iii) removal efficiency of air filters and air recirculation rate of classrooms among HVAC-and-building-related variables have the highest impacts on the risk model results in their categories. It is noticeable that changes in the total number of enrolled students alone do not influence the transmission risk in assumed microenvironment significantly, rather the drastic change in the transmission risk is driven by the variation in the ratio of the number of infected cases in the infector’s classroom to the number of enrolled students.

# **APPENDIX E**

An updatedversion of Figure 7 from the main manuscript comparing the transmission risk of measles in six typical school settings in the US among susceptible students with estimated transmission rates of measles during the first generations of infection outbreaks in schools from developed countries among susceptible students reported in existing epidemiological studies.


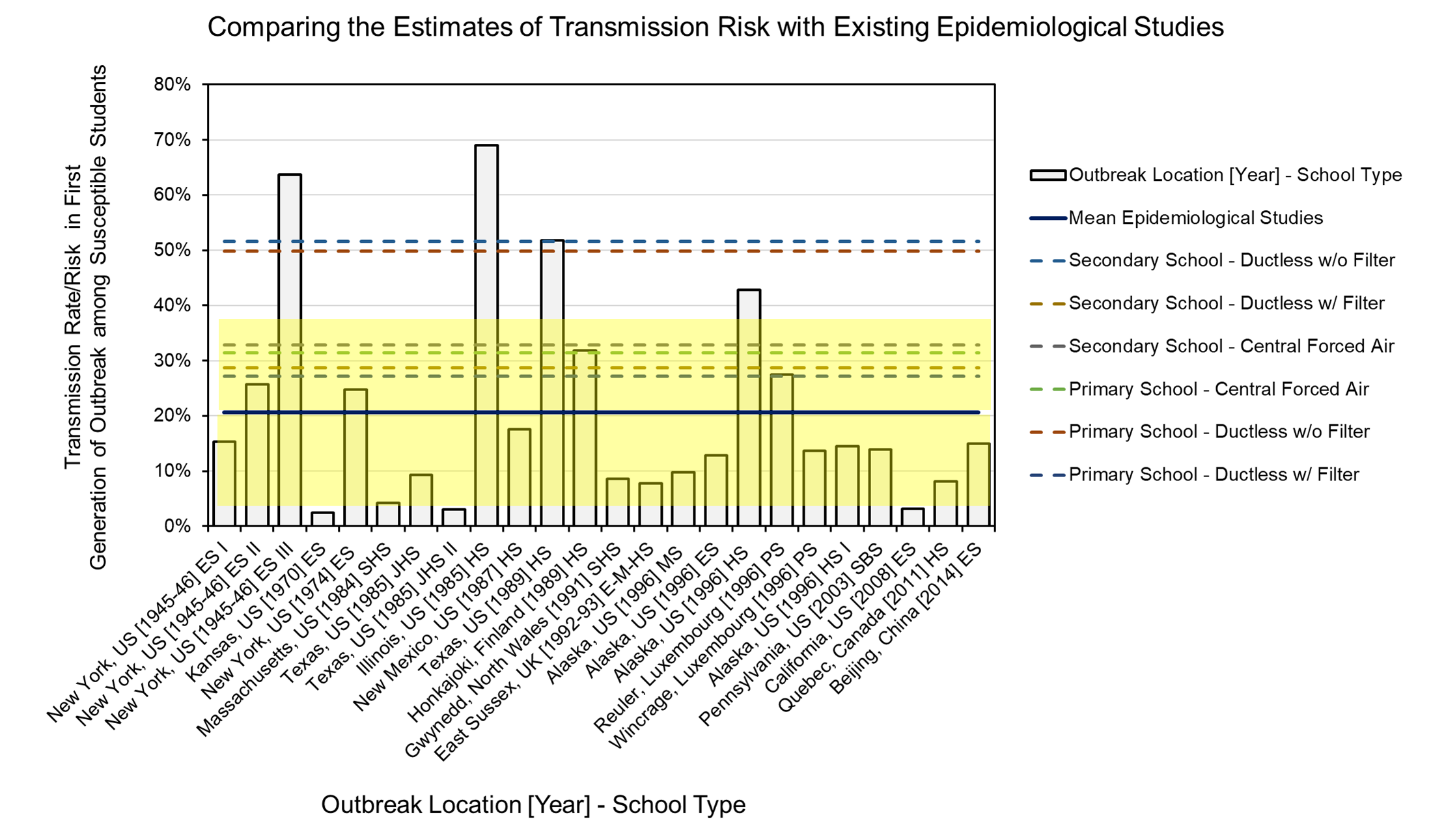


**Figure S.4.** Comparing measles transmission risk in six typical school settings in the US among susceptible students with estimated transmission rates of measles during first generations of infection outbreaks in schools from developed countriesamong susceptible students reported in existing epidemiological studies

# **REFERENCES**

1. Azimi P, Zhao D, Stephens B. Modeling the impact of residential HVAC filtration on indoor particles of outdoor origin (RP-1691). Sci Technol Built Environ. 2016 May 18;22(4):431–62.

2. Riley EC, Murphy G, Riley RL. AIRBORNE SPREAD OF MEASLES IN A SUBURBAN ELEMENTARY SCHOOL. Am J Epidemiol. 1978 May 1;107(5):421–32.

3. Chen RT, Goldbaum GM, Wassilak SGF, Markowitz LE, Orenstein WA. AN EXPLOSIVE POINT-SOURCE MEASLES OUTBREAK IN A HIGHLY VACCINATED POPULATIONMODES OF TRANSMISSION AND RISK FACTORS FOR DISEASE. Am J Epidemiol. 1989 Jan 1;129(1):173–82.

4. Azimi P, Stephens B. HVAC filtration for controlling infectious airborne disease transmission in indoor environments: Predicting risk reductions and operational costs. Build Environ. 2013 Dec 1;70:150–60.

5. Deru M, Field K, Studer D, Benne K, Griffith B, Torcellini P, et al. U.S. Department of Energy commercial reference building models of the national building stock. Publ E. 2011 Feb 1;1–118.

6. EPA U. Exposure factors handbook: 2011 edition. Intake Fish Shellfish Natl Cent Environ Assess. 2011;10:66.

7. Snyder TD, Brey C de, Dillow SA. Digest of Education Statistics 2017 53rd Edition. U.S. Department of Education, National Center for Education Statistics; 2019 Jan. Report No.: NCES 2018-070.

8. Chan TC, Jarman D. Departmentalize Elementary Schools. Principal. 2004;84(1):70–2.

9. Strohl A, Schmertzing L, Schmertzing R, Hsiao E. Comparison of self-contained and departmentalized elementary teachers’ perceptions of classroom structure and job satisfaction. J Stud Educ. 2014;4(1):109–127.

10. Chen C-S. Numbers and Types of Public Elementary and Secondary Schools From the Common Core of Data: School Year 2009–10 First Look. U.S. Department of Education, National Center for Education Statistics; 2012 Sep. Report No.: NCES 2011-345rev.

11. Moss WJ. Measles. The Lancet. 2017 Dec 2;390(10111):2490–502.

12. Moss WJ, Griffin DE. Global measles elimination. Nat Rev Microbiol. 2006 Dec;4(12):900–8.

13. Perry RT, Halsey NA. The Clinical Significance of Measles: A Review. J Infect Dis. 2004 May 1;189(Supplement_1):S4–16.

14. CDC. Measles: Answers to Common Questions [Internet]. Centers for Disease Control and Prevention. 2019 [cited 2019 Aug 8]. Available from: https://www.cdc.gov/measles/about/faqs.html

15. Hill HA. Vaccination Coverage Among Children Aged 19–35 Months — United States, 2017. MMWR Morb Mortal Wkly Rep [Internet]. 2018 [cited 2019 Aug 7];67. Available from: https://www.cdc.gov/mmwr/volumes/67/wr/mm6740a4.htm

16. Centers for Disease Control and Prevention (CDC). National, state, and urban area vaccination coverage levels among children aged 19-35 months--United States, 2000. MMWR Morb Mortal Wkly Rep. 2001 Aug 3;50(30):637–41.

17. Walker TY, Elam-Evans LD, Yankey D, Markowitz LE, Williams CL, Mbaeyi SA, et al. National, Regional, State, and Selected Local Area Vaccination Coverage Among Adolescents Aged 13–17 Years — United States, 2017. Morb Mortal Wkly Rep. 2018 Aug 24;67(33):909–17.

18. Reagan-Steiner S. National, Regional, State, and Selected Local Area Vaccination Coverage Among Adolescents Aged 13–17 Years — United States, 2015. MMWR Morb Mortal Wkly Rep [Internet]. 2016 [cited 2019 Aug 8];65. Available from: https://www.cdc.gov/mmwr/volumes/65/wr/mm6533a4.htm

19. Reagan-Steiner S, Yankey D, Jeyarajah J, Elam-Evans LD, Singleton JA, Curtis CR, et al. National, Regional, State, and Selected Local Area Vaccination Coverage Among Adolescents Aged 13–17 Years — United States, 2014. MMWR Morb Mortal Wkly Rep. 2015 Jul 31;64(29):784–92.

20. Lopez AS, Cardemil C, Pabst LJ, Cullen KA, Leung J, Bialek SR. Two-Dose Varicella Vaccination Coverage Among Children Aged 7 years — Six Sentinel Sites, United States, 2006–2012. MMWR Morb Mortal Wkly Rep. 2014 Feb 28;63(8):174–7.

21. Landen MG, Beller M, Funk E, Rolka H, Middaugh J. Measles outbreak in Juneau, Alaska, 1996: implications for future outbreak control strategies. Pediatrics. 1998;102(6):E71.

22. Choi YH, Gay N, Fraser G, Ramsay M. The potential for measles transmission in England. BMC Public Health. 2008 Sep 26;8(1):338.

23. Brunell PA, Weigle K, Murphy MD, Shehab Z, Cobb E. Antibody Response Following Measles-Mumps-Rubella Vaccine Under Conditions of Customary Use. JAMA. 1983 Sep 16;250(11):1409–12.

24. Hayden GF. Clinical Review : Measles Vaccine Failure: A Survey of Causes and Means of Prevention. Clin Pediatr (Phila). 1979 Mar 1;18(3):155–6.

25. Nkowane BM, Bart SW, Orenstein WA, Baltier M. Measles outbreak in a vaccinated school population: epidemiology, chains of transmission and the role of vaccine failures. Am J Public Health. 1987 Apr 1;77(4):434–8.

26. Weibel RE, Buynak EB, McLean AA, Hilleman MR. Follow-Up Surveillance for Antibody in Human Subjects following Live Attenuated Measles, Mumps, and Rubella Virus Vaccines. Proc Soc Exp Biol Med. 1979 Nov 1;162(2):328–32.

27. Schools and Staffing Survey (SASS) [Internet]. [cited 2019 Sep 18]. Available from: https://nces.ed.gov/surveys/sass/tables/sass1112_2013312_s2s_007.asp

28. Hanna J, Richards A, Young D, Hills S, Humphreys J. Measles in health care facilities: some salutary lessons. Commun Dis Intell. 2000;24(7):211–212.

29. Remington PL, Hall WN, Davis IH, Herald A, Gunn RA. Airborne Transmission of Measles in a Physician’s Office. JAMA. 1985 Mar 15;253(11):1574–7.

30. Bloch AB, Orenstein WA, Ewing WM, Spain WH, Mallison GF, Herrmann KL, et al. Measles Outbreak in a Pediatric Practice: Airborne Transmission in an Office Setting. Pediatrics. 1985 Apr 1;75(4):676–83.

31. Schools and Staffing Survey (SASS) [Internet]. [cited 2019 Sep 18]. Available from: https://nces.ed.gov/surveys/sass/tables/sass0708_035_s1s.asp

32. Conklin MT, Lambert LG. Eating at school: A summary of NFSMI research on time required by students to eat lunch. Natl Food Serv Manag Inst Univ Mississippi2001. 2001;

33. DOE IEA. 2003 Commercial Building Energy Consumption Survey. Washington, DC: DOE Energy Information Administration; 2003.

34. Winiarski DW, Jiang W, Halverson MA. Review of Pre- and Post-1980 Buildings in CBECS - HVAC Equipment [Internet]. Pacific Northwest National Lab. (PNNL), Richland, WA (United States); 2006 Dec [cited 2019 Aug 6]. Report No.: PNNL-20346. Available from: https://www.osti.gov/biblio/1013959

35. DOE IEA. 2012 Commercial Building Energy Consumption Survey. Washington, DC: DOE Energy Information Administration; 2012.

36. Winiarski DW, Halverson MA, Butzbaugh JB, Cooke AL, Bandyopadhyay GK, Elliott DB. Analysis for Building Envelopes and Mechanical Systems Using 2012 CBECS Data. Pacific Northwest National Lab.(PNNL), Richland, WA (United States); 2018.

37. Rowand C. How old are America’s public schools. Educ Stat Q. 1999;1(1):53–56.

38. Alexander D, Lewis L. Condition of America’s Public School Facilities: 2012-13. First Look. NCES 2014-022. Natl Cent Educ Stat. 2014;

39. Polidori A, Fine PM, White V, Kwon PS. Pilot study of high-performance air filtration for classroom applications. Indoor Air. 2013;23(3):185–95.

40. Chan WR, Parthasarathy S, Fisk WJ, McKone TE. Estimated effect of ventilation and filtration on chronic health risks in U.S. offices, schools, and retail stores. Indoor Air. 2016;26(2):331–43.

41. ASHRAE S. Standard 62.1-2016 Ventilation for Acceptable Indoor Air Quality. Am Soc Heat. 2016;

42. NAFA. Filtration for Schools. Madison, WI: National Air Filtration Association; 2012.

43. EPA. Indoor Air Quality Tools for Schools Coordinator’s Guide A Guide to Implementing an IAQ Program. U.S. Environmental Protection Agency; 2009 Jan.
